# In vivo inhibition of nuclear ACE2 translocation protects against SARS-CoV-2 replication and lung damage through epigenetic imprinting

**DOI:** 10.1038/s41467-023-39341-4

**Published:** 2023-06-27

**Authors:** Wen Juan Tu, Michelle Melino, Jenny Dunn, Robert D. McCuaig, Helle Bielefeldt-Ohmann, Sofiya Tsimbalyuk, Jade K. Forwood, Taniya Ahuja, John Vandermeide, Xiao Tan, Minh Tran, Quan Nguyen, Liang Zhang, Andy Nam, Liuliu Pan, Yan Liang, Corey Smith, Katie Lineburg, Tam H. Nguyen, Julian D. J. Sng, Zhen Wei Marcus Tong, Keng Yih Chew, Kirsty R. Short, Roger Le Grand, Nabila Seddiki, Sudha Rao

**Affiliations:** 1 Gene Regulation and Translational Medicine Laboratory, QIMR Berghofer Medical Research Institute, Brisbane, QLD Australia; 2 School of Chemistry and Molecular Biosciences, The University of Queensland, Brisbane, QLD Australia; 3 Australian Infectious Diseases Research Centre, The University of Queensland, Brisbane, QLD Australia; 4 School of Biomedical Sciences, Charles Sturt University, Wagga Wagga, NSW 2678 Australia; 5 Genomics and Machine Learning Lab, Division of Genetics and Genomics, Institute for Molecular Bioscience, University of Queensland, Brisbane, QLD 4072 Australia; 6 NanoString Technologies Inc., Seattle, WA 98109 USA; 7 Translational and Human Immunology Laboratory, QIMR Berghofer Medical Research Institute, Brisbane, QLD Australia; 8 Flow and Imaging Facility, QIMR Berghofer Medical Research Institute, Brisbane, QLD Australia; 9 Australian Infectious Diseases Research Centre, Global Virus Network Centre of Excellence, Brisbane, QLD Australia; 10 Université Paris-Saclay, INSERM U1184, CEA, Center for Immunology of Viral, Auto-immune, Hematological and Bacterial diseases (IMVA-HB/IDMIT), Fontenay-aux-Roses, France

**Keywords:** Molecular medicine, Epigenetics

## Abstract

In vitro, ACE2 translocates to the nucleus to induce SARS-CoV-2 replication. Here, using digital spatial profiling of lung tissues from SARS-CoV-2-infected golden Syrian hamsters, we show that a specific and selective peptide inhibitor of nuclear ACE2 (NACE2i) inhibits viral replication two days after SARS-CoV-2 infection. Moreover, the peptide also prevents inflammation and macrophage infiltration, and increases NK cell infiltration in bronchioles. NACE2i treatment increases the levels of the active histone mark, H3K27ac, restores host translation in infected hamster bronchiolar cells, and leads to an enrichment in methylated ACE2 in hamster bronchioles and lung macrophages, a signature associated with virus protection. In addition, ACE2 methylation is increased in myeloid cells from vaccinated patients and associated with reduced SARS-CoV-2 spike protein expression in monocytes from individuals who have recovered from infection. This protective epigenetic scarring of ACE2 is associated with a reduced latent viral reservoir in monocytes/macrophages and enhanced immune protection against SARS-CoV-2. Nuclear ACE2 may represent a therapeutic target independent of the variant and strain of viruses that use the ACE2 receptor for host cell entry.

## Abstract (web summary)

The entry receptor for SARS-CoV-2, ACE2, translocates to the nucleus following infection with this virus. Here, the authors demonstrate that a peptide targeting the ACE2 nuclear localization signal promotes its methylation and abrogates viral replication of SARS-CoV-2 in Syrian hamsters.

## Introduction

The successful development and rollout of COVID-19 vaccines was a positive outcome from the pandemic. Nevertheless, vaccine efficacy can quickly wane, especially in the face of new variants. Therapies that complement vaccine-induced immune protection are still needed, especially for vulnerable individuals.

Most proteins undergo post-translational modifications (PTMs), and epigenetic enzymes fine-tune critical protein function in response to environmental cues via methylation and demethylation PTMs and histone PTMs that modify chromatin structure^1–3^. We previously demonstrated that ACE2 is methylated and demethylated at lysine 31 and that glutamine 493 in the SARS-CoV-2 spike protein receptor-binding domain (RBD) binds to this residue^4^. In vitro, SARS-CoV-2 infection decreased ACE2 methylation, and ACE2 lysine 31 hypermethylation significantly decreased interactions with the SARS-CoV-2 spike protein^4^.

SARS-CoV-2 is a single-stranded, positive-sense RNA virus. Like some other RNA viruses, SARS-CoV-2 exploits the importin nuclear shuttling machinery during infection to hijack host cell transcription and responses^5,6^. Several studies have now shown that targeting the importin-mediated nuclear transport machinery may be critical for inhibiting SARS-CoV-2 infection. Importin-α (IMPα) plays a role in SARS-CoV-2 infection through nucleocytoplasmic shuttling of the SARS-CoV nucleocapsid protein and subsequent host cell division^7–11^.

We previously discovered a highly conserved nuclear localization signal (NLS) within the C-terminal (cytoplasmic) tail of ACE2^4^. ACE2, via its interaction with IMPα, translocates to the nucleus following SARS-CoV-2 infection. The crystal structure of the ACE2 cytoplasmic tail- IMPα complex revealed a direct interaction between the lysine residues within the NLS motif of ACE2 and IMPα, as confirmed by microscale thermophoresis and fluorescence polarization^4^. Exploiting this knowledge, we developed a cell permeable and highly selective ACE2-targeting peptide, NACE2i, which spans and targets the ACE2 NLS motif. NACE2i successfully abolishes nuclear translocation of ACE2 and viral replication in human cell lines infected with SARS-CoV-2^4^. The nuclear (n)ACE2-induced transcriptional program is an early event critical for SARS-CoV-2 replication.

Here, we extend these in vitro studies and address the impact of targeting the nACE2 pathway with NACE2i in the golden Syrian hamster model of SARS-CoV-2 infection, which closely mimics mild, resolving COVID-19 disease in humans^12^. We show that targeting the nACE2 pathway significantly inhibits viral replication and prevents early lung inflammation and pathology associated with COVID-19. Using digital spatial analysis, we identify a transcriptional signature associated with SARS-CoV-2 infection and NACE2i-induced relief of inflammatory lung pathology. NACE2i induces a protective epigenetic ACE2 methylation signature in the bronchiolar epithelium of infected hamsters and in monocytes from blood from vaccinated individuals. This protective epigenetic scarring of ACE2 is associated with a reduced latent viral reservoir in monocytes/macrophages and enhanced immune protection against SARS-CoV-2. Therapeutics that target nuclear ACE2 represent a promising antiviral treatment independent of the variant and strain of viruses that use the ACE2 receptor for host cell entry.

## Results

### NACE2i selectively targets nuclear ACE2 following SARS-CoV-2 infection

**Fig. 1:**
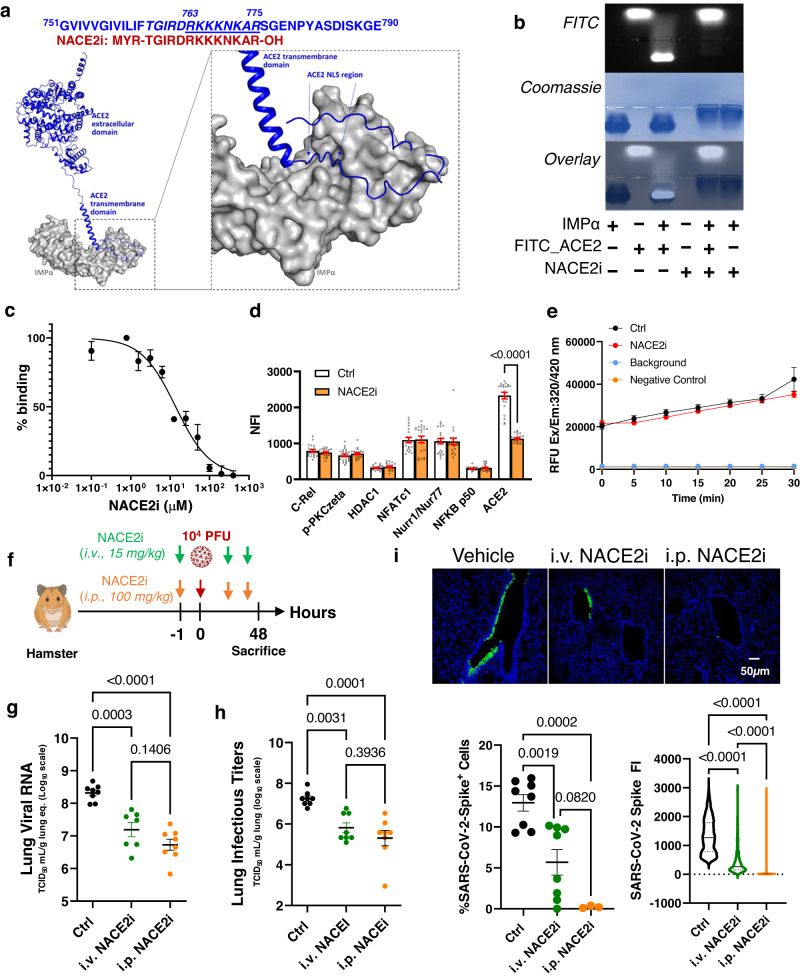
NACE2i selectively targets nuclear ACE2 following SARS-CoV-2 infection and inhibits viral replication in hamster lungs. **a** Using NLS prediction software (NLStradamus, Revision r.9), an NLS motif (italics) and critical NLS residues were identified (underlined) at the ACE2 C-terminus. A peptide inhibitor targeting this region was designed (red). AlphaFold docked the NLS region of ACE2 in the same location observed in the crystal structure^4^, demonstrating accessibility between the transmembrane region and the cytoplasmic NLS. **b** Electrophoresis mobility shift assay confirming the IMP1α and ACE2 interaction via the C-terminal domain. The ACE2 C-terminal domain is FITC-labeled. *n* = 2 experimental replicates. **c** Microscale thermophoresis and fluorescence polarization to assess NACE2i inhibition of binding between IMP1α and ACE2. *n* = 3 experimental replicates, KD shown represent the mean ± SD. **d** Cells immunostained with NFACT1, C-Rel, ACE2, pPKC-zeta, Nurr1/Nur77, HDAC1 or NFKB p50. Nuclear fluorescent intensity (NFI) was determined using ImageJ analysis; *n* = 2 technical replicates (*n* = 20 cells analyzed from 1 field of view (FOV) per group). Data are mean ± SEM. One-way ANOVA with Bonferroni post hoc test. **e** ACE2 activity was measured in Caco2 cell lysates ± NACE2i. Data represent mean ± SEM, *n* = 3 technical replicates. **f** Scheme of SARS-CoV-2 infection in golden Syrian hamsters. All the hamsters were sacrificed at day 2 post-infection (dpi), lung tissues were collected (*n* = 8 hamsters/group in a single experiment). **g** qRT-PCR analysis of SARS-CoV-2 RNA in infected lungs from golden Syrian hamsters treated ± NACE2i. RNA yield is presented as log10 TCID_50_ eq./ml. Data represent mean ± SEM, *n* = 7 animals, i.v. NACE2i; *n* = 8 animals, Ctrl, i.p. NACE2i. One-way ANOVA with Tukey’s post hoc test. **h** TCID_50_ assay to measure infectious viral titers in lungs of infected hamsters. Data represent mean ± SEM, *n* = 5 animals/group. One-way ANOVA with Tukey’s post hoc test. **i** Representative images of lungs from SARS-CoV-2-infected hamsters treated with either vehicle control, i.p. NACE2i, or i.v. NACE2i. SARS-CoV-2 spike protein (green), DAPI (blue) for nuclei. Graphs depict the population dynamics of SARS-CoV-2 spike positive cells and the fluorescent intensity of the SARS-CoV-2 spike protein (5 FOVs/animal from *n* = 8 animals, Ctrl, i.v. NACE2i; *n* = 3 animals, i.p. NACE2i). Data represent mean ± SEM. One-way ANOVA with Tukey’s post hoc test.

Some cell membrane receptors can translocate to the nucleus to regulate chromatin remodeling^13–17^. We previously showed that SARS-CoV-2 infection translocates ACE2 to the nucleus and that targeting this translocation with the NACE2i peptide significantly inhibits viral replication in human cells^4^. The target sequence of the ACE2 NLS and the NACE2i sequence are shown in Fig. 1a. The crystal structure of the ACE2 cytoplasmic tail-IMPα complex revealed a direct interaction between ACE2 NLS motif lysine residues and IMPα. For further evidence of interaction, we used AlphaFold 2 to perform structural analysis of IMPα complexed with ACE2 residues 721-805, comprising the extracellular domain, transmembrane domain, and intracellular domain containing the NLS, which correctly placed the ACE2 NLS binding sequence in the major, crystal-determined site of IMPα and confirmed accessibility between the transmembrane region and the NLS (Fig. 1a). Pull down of ACE2 protein from Caco2 cell lysates and immunoblotting with antibodies targeting IMPα1 showed that ACE2 and IMPα are in complex (Supplementary Fig. 1a).

We next confirmed that NACE2i inhibited nuclear shuttling of ACE2 by directly inhibiting the ACE2-IMPα complex in vitro (Fig. 1b, c). NACE2i specifically targets nuclear ACE2 and not other nuclear proteins targeted by the importin pathway (Fig. 1d and ref. ^4^). An ACE2 activity assay showed that NACE2i has no impact on its enzymatic activity, indicating that ACE2 can still function as a renin-angiotensin system regulator^18^ (Fig. 1e). The proximity ligation assay (PLA) detects close interactions between two protein targets, so using PLA with antibodies targeting the NLS region of ACE2 and IMPα in H1299 non-small cell lung carcinoma cells treated with either control or increasing concentrations of NACE2i, NACE2i significantly inhibited ACE2-IMPα interactions in a dose-dependent manner (Supplementary Fig. 1b). Confirming our previous findings that NACE2i can significantly inhibit SARS-CoV-2 replication in Caco2 cells^4^, virus replication was reduced in IMPα knockdown cells (Supplementary Fig. 1c). Collectively, these data and our previous work^4^ show that NACE2i is a selective inhibitor of the ACE2-IMPα interactions critical for SARS-CoV-2 replication.

### Safety profile of NACE2i in pre-clinical mouse and hamster animal models of COVID-19

To assess the in vivo safety of NACE2i, female C57BL/6 J mice were treated with a single bolus dose of 3, 10, 30, or 100 mg/kg NACE2i intraperitoneally (i.p.) and were monitored daily for seven days. NACE2i treatment was tolerated at doses up to 100 mg/kg without significant changes in body weight (Supplementary Fig. 1d; Supplementary Table 1). In a second NACE2i toxicity study, male C57BL/6 J mice were treated with daily boluses of either saline vehicle or NACE2i at 3, 10, and 30 mg/kg intravenously (i.v.) for six days, and i.v. NACE2i was tolerated up to 30 mg/kg without significant changes in body or organ weight (Supplementary Fig. 1e, f; Supplementary Table 1).

Escalating doses of i.p. NACE2i (25, 50, 100 mg/kg) were also administered daily to golden Syrian hamsters. NACE2i was well tolerated at doses up to 100 mg/kg without a decrease in body weight, minimal changes in body temperature, and no observable discomfort (Supplementary Fig. 1g) throughout the study. NACE2i was also stable in plasma from male cynomolgus monkeys, with a T_1/2_ of 867.2 min. 78% of NACE2i was retained in the plasma after 360 min incubation, while the control compound somatostatin acetate decreased to 3.8% and procaine hydrochloride to 0.0% (Supplementary Fig. 1h, i).

### NACE2i treatment inhibits viral replication in the lungs of golden Syrian hamsters

Golden Syrian hamsters are used as models of human COVID-19^12,19^. In previous studies, SARS-CoV-2 viral titers peaked in hamster lungs two days after inoculation (days post-inoculation; dpi) followed by rapid viral clearance by day seven^12^. Therefore, here we focused on early infection at day two post-infection. Animals were treated with i.v. or i.p. NACE2i one hour before SARS-CoV-2 infection (10^4^ PFU; intranasal administration) (Fig. 1f). The decrease in mean body weights of i.v. and i.p. NACE2i-treated animals was lower than vehicle controls at 1 and 2 dpi (Supplementary Fig. 1j), and there were significant reductions in viral RNA in the lungs of animals treated with i.v. and i.p. NACE2i (Fig. 1g). When normalized to vehicle controls, both i.v. and i.p. NACE2i-treated animals showed significant decreases in viral RNA of 88% and 96%, respectively (Supplementary Fig. 1k). Lung infectious titers, as measured by the TCID_50_, were significantly reduced in i.v. and i.p. NACE2i-treated animals compared with vehicle controls (Fig. 1h), with us electing to test both the i.v. and i.p. routes to demonstrate comparable efficacy irrespective of the delivery route with a view to eventual clinical translation. Furthermore, control peptides not targeting the NLS region of ACE2 (i.v. ACE2i at doses of 10, 30, and 100 mg/kg) were similarly well tolerated in male C57BL/6 J mice (Supplementary Fig. 1l, m) and did not affect body weight reductions (Supplementary Fig. 1n), viral RNA yield, viral load (%), or lung infectious titers (Supplementary Fig. 1o) in golden hamsters. Digital pathology imaging demonstrated that i.p. and i.v. NACE2i significantly reduced the population of SARS-CoV-2 spike protein-positive cells in the bronchiolar epithelium and, even when cells were positive for SARS-CoV-2 spike protein, the overall signal intensity was significantly reduced by i.p. and i.v. NACE2i (Fig. 1i).

### NACE2i treatment reduces lung inflammation and induces high perforin expression in CD3^+^ lymphocytes

**Fig. 2:**
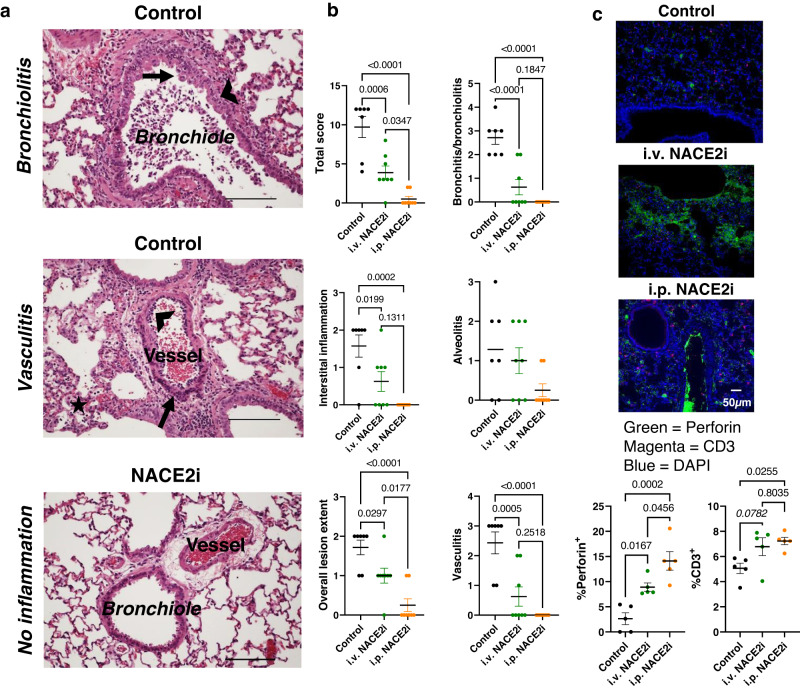
NACE2i treatment improves inflammatory lung pathology and induces a protective perforin signature in CD3^+^ lymphocytes. **a** Representative H&E-stained tissue sections illustrating the features of lungs from vehicle (*n* = 7 animals) and NACE2i-treated animals (*n* = 8 animals). Top panel: bronchiolitis with degeneration, necrosis (arrow), and exfoliation of epithelial cells accompanied by transmural leukocyte infiltration (arrowhead). Middle panel: vasculitis characterized by margination (arrowhead) and transmural migration (arrow) of heterophils and monocytes accompanied by endothelial cell and smooth muscle cell damage (arrow). Also seen in this panel is alveolar macrophage and interstitial leukocyte accumulation (star). Bottom panel: no inflammation observed in the vessels or bronchioles. Scale bar, 200 μm. **b** Histopathological scoring of whole infected lungs from H&E-stained tissue sections in (**a**). Note: an additional score of zero was recorded for pneumocyte hyperplasia for all samples. Data represent mean ± SEM, *n* = 7 animals, Control; n = 8 animals, i.v. NACE2i and i.p. NACE2i. One-way ANOVA with Tukey’s post hoc test. **c** Representative images (scale bar 50 µm) of FFPE lung sections from SARS-CoV-2-infected golden Syrian hamsters treated with either vehicle control, i.p. NACE2i, or i.v. NACE2i. FFPE lung tissues were stained for perforin (green) and CD3 (magenta). DAPI (blue) was used to stain nuclei. Analysis of the population dynamics of CD3- and perforin-positive cells were carried out using ASI Digital Pathology (at least 5 FOVs/animal from *n* = 5 animals/group). Data represent mean ± SEM. One-way ANOVA with Tukey’s post hoc test.

To assess the impact of NACE2i treatment on SARS-CoV-2-induced lung pathology, lung sections from infected golden hamsters were scored with respect to overall lesion extent, bronchitis, alveolitis, vasculitis, interstitial inflammation, and pneumocyte hyperplasia by a single veterinary pathologist blinded to the treatments (Fig. 2a)^19^. Individual parameters were scored using 0-4 or 0-5 scales, and scores for each lung were summed to obtain a total histopathological score (Fig. 2b). At the early stages of infection at 2 dpi, histopathological examination revealed mild to moderate pathological changes in SARS-CoV-2-infected hamster lungs (Fig. 2a, b). There was marked bronchiolitis with severe degenerative necrosis of bronchiolar epithelial cells and intraepithelial leukocytosis with widespread apoptosis (Fig. 2a, top panel; Fig. 2b). Early vascular changes in the vehicle group included margination of heterophils and monocytes with some transmural migration resulting in disruption of the endothelial lining and the smooth muscle of the media. Also, there was a marked alveolar macrophage and interstitial leukocyte infiltrate (Fig. 2a middle panel; Fig. 2b). In comparison, the majority of i.v. and i.p. NACE2i-treated animals displayed minimal alveolar, bronchiolar, or vascular changes (Fig. 2a bottom panel; Fig. 2b). Mean cumulative scores were substantially lower in treated animals than the vehicle group (Fig. 2b).

There was peri-bronchiolar accumulation of CD3^+^ T cells in infected hamsters at 5 dpi, probably to facilitate the rapid clearance of infected cells^12^. COVID-19 patients, particularly those with severe COVID-19, show impaired antiviral responses with decreased CD3^+^ T cells and NK cells, reduced perforin and granzyme A expression, and increased IL-6 serum levels^20^. Treatment with i.v. or i.p. NACE2i significantly increased the proportion of perforin-positive cells and the number of CD3^+^ cells (Fig. 2c), essential for anti-viral activity. NACE2i also reduced IL-6 expression and increased perforin expression in PBMCs isolated from a patient with severe COVID-19 (Supplementary Fig. 2).

### NACE2i treatment downregulates TLR-4 mediated inflammatory signaling and restores host translation in hamsters

To investigate tissue-specific transcriptomic signatures and viral load, we performed NanoString GeoMx Digital Spatial Profiling (DSP) on lung tissues from SARS-CoV-2 infected hamsters at 2 dpi with or without NACE2i treatment. For each tissue section, regions of interest (ROIs) were selected based on pan-cytokeratin and SARS-CoV-2 spike protein expression followed by next-generation sequencing (NGS) on the Illumina NextSeq 500 platform.

As this was the first application of the GeoMx mouse whole transcriptome atlas (WTA) to the hamster model, assay quality and sensitivity analyses were performed prior to data analysis. In-depth analyses of > 21,000 genes (WTA probe set and COVID-19 spike-in panel) across 48 selected ROIs revealed that all ROIs were above 50% sequencing saturation, and 19,966 target genes were detected. Data were further normalized using the third quartile (Q3) to identify 8431 genes expressed above LOQ2 in at least 1% of all ROIs used for downstream analysis (Supplementary Fig. 3a).

**Fig. 3:**
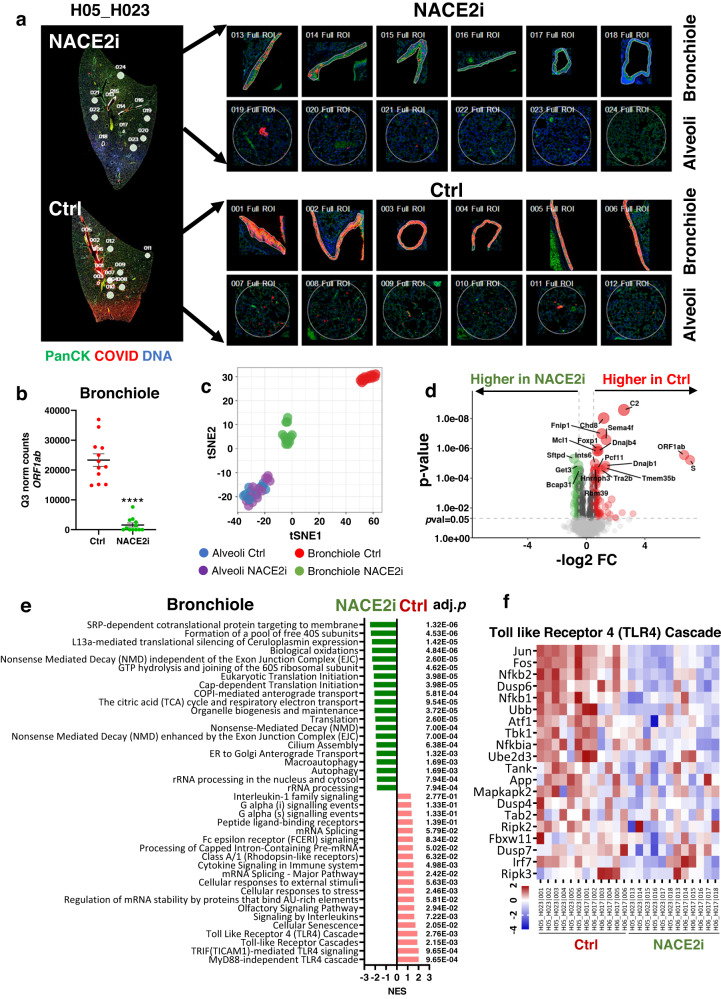
Digital spatial analysis of lungs in SARS-CoV-2-infected hamsters with and without NACE2i treatment showing inhibition of the TLR4 cascade and restoration of the host translational program. **a** Representative regions of interest (ROIs) in the lungs of SARS-CoV-2-infected control (*n* = 1 animal) and i.v. NACE2i-treated hamsters (*n* = 1 animal). Left: multi-color immunofluorescent staining with collection of ROIs. Right: ROIs selected from bronchiolar (*n* = 6 regions in each animal, top) and alveolar (*n* = 6 regions in each animal, bottom) regions in each group. PanCK/green, COVID-19/red, DNA Syto 13/blue. **b** Dot plot of Q3-normalized *ORF1ab* gene expression with *n* = 12 ROIs from *n* = 2 hamsters per group shown in bronchiolar regions of (**a**) and Supp. Fig. 3b). Shown as mean ± SEM using two-sided Welch’s *t*-test. **c** t-distributed stochastic neighbor embedding (t-SNE) clustering analysis of transcriptional profiles of ROIs from the lung bronchioles (*n* = 12 regions in two animals) and alveoli (*n* = 12 regions in two animals) in SARS-CoV-2-infected control and NACE2i-treated hamsters. **d** Identification of differentially expressed genes (DEGs) in the lung bronchioles of SARS-CoV-2-infected control and NACE2i-treated hamsters (*n* = 2 hamsters/group). Horizontal dashed line, *p* = 0.05; vertical dashed lines, log_2_(fold change) = 0.5, fold change. DEGs were defined as *p* = 0.05 and log2-fold change of 0.5. Top 20 genes are marked and DEGs are colored. **e** Gene Set Enrichment Analysis (GSEA) of top 20 pathways enriched in the lung bronchioles of SARS-CoV-2-infected control and NACE2i-treated hamsters (*n* = 2 hamsters/group). Adjusted p-values are shown on the right. **f** Heat map of the top 20 genes in the toll-like receptor 4 (TLR4) cascade. Shown as z-scores. *n* = 2 hamsters/group.

Consistent with previous findings^21^, only bronchiolar regions, and not alveolar regions, showed high levels of SARS-CoV-2 spike protein expression, as expected for early-stage infection in hamsters at 2 dpi (Fig. 3a, Supplementary Fig. 3b). Since both i.p. and i.v. NACE2i significantly reduced SARS-CoV-2, we combined data from the two NACE2i treatments (*n* = 2) and two controls (*n* = 2) for transcription analysis. NACE2i treatment significantly reduced the expression of SARS-CoV-2 *ORF1ab* in mainly bronchiolar regions but also alveoli compared with controls (Fig. 3b, Supplementary Fig. 3c). tSNE clustering of all ROIs highlighted strong separation between clusters derived from bronchiolar controls and NACE2i-treated bronchioles but not alveoli **(**Fig. 3c). Differential gene expression analysis between bronchiole clusters revealed downregulation of several genes in the NACE2i-treated group including *C2* of the complement cascade and *DnaJb1* and *DnaJb4* (DnaJ heat shock protein family (Hsp40) member B1 and B4), which are involved in virus replication, hyperinflammation, and tissue damage during viral infections (Fig. 3d). Although only a few genes were differentially expressed in alveoli, both viral *ORF1ab* and S genes were significantly downregulated in NACEi2-treated hamsters (Supplementary Fig. 3d).

Finally, GSEA of bronchiole ROIs confirmed that the top enriched pathways were all related to inflammatory signaling in control lungs, including the My88-independent TLR4 cascade, TRIF (TICAM1)-mediated TLR4 signaling, toll-like receptor cascades, and the toll-like receptor 4 (TLR4) cascade (Fig. 3e). TLR4 activation by SARS-CoV-2 infection has been suggested to increase surface ACE2 expression to facilitate viral entry and contribute to hyperinflammation to cause multi-organ failure in severe COVID-19 patients^22^. Indeed, the top 20 genes in the TLR4 pathway were downregulated in NACE2i-treated hamsters compared with controls (Fig. 3f). Furthermore, GSEA analysis of bronchiole ROIs revealed that NACE2i treatment regulated key pathways involved in nuclear events and translation such as ribosomal RNA formation, nonsense-mediated decay (NMD), and translation initiation (Fig. 3e). The same key pathways were also enriched in the alveoli of NACE2i-treated lungs (Supplementary Fig. 3e). It has been shown that SARS-CoV-2 suppresses the host anti-viral response by disrupting essential cellular activities including mRNA splicing, ribosome binding, and protein translation and trafficking^23^. Since NACEi2 inhibits nuclear ACE2 translocation, nuclear ACE2 may be required for interactions between SARS-CoV-2 proteins and human RNA.

### NACE2i decreases macrophages and induces NK cells and ACE2 methylation in bronchiolar cells in SARS-CoV-2-infected golden Syrian hamsters

**Fig. 4:**
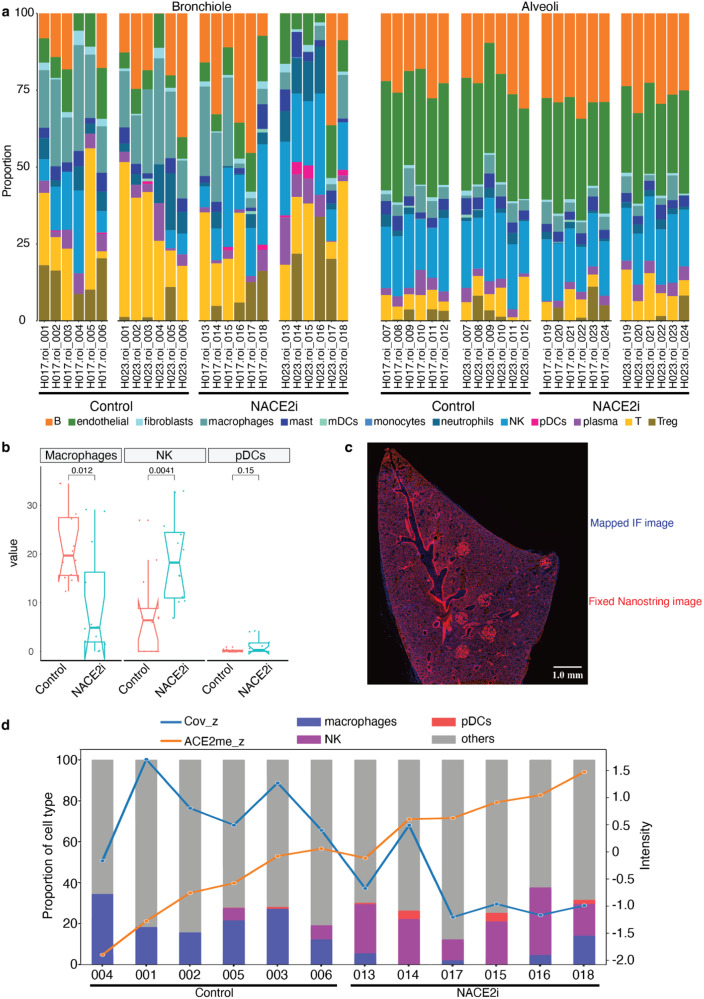
Targeting nuclear ACE2 with NACE2i leads to inhibition of SARS-CoV-2 spike protein, pro-inflammatory macrophages with simultaneous induction of NK cells, and ACE2^me^ in treated lung tissues. **a** Stacked bar plot showing cell type deconvolution results for NanoString DSP data. ROIs are split by tissue type (bronchiole (left) and alveoli (right)) and ordered by control and NACE2i-treated samples in each tissue type. Each bar indicates the proportion of each cell type in a particular ROI, *n* = 12 ROIs from *n* = 2 hamsters per group. **b** Notched box plot showing the proportion of macrophages, NK cells, and pDCs for control and NACE2i-treated bronchioles using NanoString DSP data (*n* = 12 ROIs from *n* = 2 hamsters per group). The Wilcoxon test was performed. The notches show the 95% confidence interval estimates of the median. The interquartile range (IQR) covers the middle 50% of the distribution. The vertical lines show the range, extended to a maximum of 1.5x IQR beyond the box. **c** Overlaying of registered IF images (in blue, appearing as a faint layer on top of the red background) onto NanoString images (in red). The overlayed images show that the two layers are well aligned after image registration (*n* = 1 hamster/group). **d** Integrated comparison of IF data and NanoString data for 12 ROIs in sectionH05_H023 (*n* = 1 hamster/group). Line plot shows the expression of SARS-CoV-2 spike protein and ACE2^me^ from IF data. Stacked bar plot shows cell type proportions from NanoString DSP data. x-axis shows ROIs, the left y-axis shows cell proportion, and the right y-axis shows expression values of the two markers (on z-scale).

Previous studies have shown that the accumulation of long-lived macrophages in the lungs of COVID-19 patients mediates inflammasome activation and is associated with pulmonary fibrosis^24,25^. Moreover, persistent dysfunction of natural killer (NK) cells and dendritic cells has been reported in severe COVID-19, indicating impaired anti-SARS-CoV-2 activity^26–29^. Cell type deconvolution analysis of NanoString DSP data showed that NACE2i significantly decreased macrophages and increased NK cells in the bronchiolar regions of infected hamsters, indicating that NACE2i reduces inflammation and induces anti-viral responses (Fig. 4a, b).

To explore SARS-CoV-2-mediated regulation of ACE2 PTMs, we developed a custom ACE2^me^ antibody (Supplementary Fig. 4a) and validated its specificity in Caco2 cells (Supplementary Fig. 4c). As expected, ACE2^me^ was detected by immunoblotting in ACE2 pull downs from Caco2 cells (Supplementary Fig. 4b). MRC5 cells are resistant to SARS-CoV-2 infection due to a lack of ACE2 expression^30,31^. We further validated the specificity of the custom ACE2^me^ antibody by transfecting MRC5 cells with plasmid vector only, wild-type ACE2 (ACE2_WT), and a lysine to alanine mutant ACE2 (ACE2_K31A) (Supplementary Fig. 4d), with ACE2 and ACE2^me^ expression confirmed by immunofluorescence. The ACE2^me^ antibody only detected signal in ACE2_WT but not ACE2_K31A mutant cells, while antibodies targeting ACE2 detected signal in both wild-type and mutant MRC5 cells (Supplementary Fig. 4e).

We extensively optimized ACE2 and ACE2^me^ antibody staining in hamster lung tissues (Supplementary Fig. 4f) and examined the temporospatial expression of ACE2^me^ in infected hamster lungs. Image registration was used for immunofluorescence and NanoString DSP data integration (Fig. 4c and Supplementary Fig. 5a, b), which revealed that ACE2^me^ expression was higher in bronchiolar areas without SARS-CoV-2 positivity, suggesting a negative correlation between ACE2^me^ expression and viral burden in infected hamster lungs (Fig. 4d and Supplementary Fig. 5c).

**Fig. 5:**
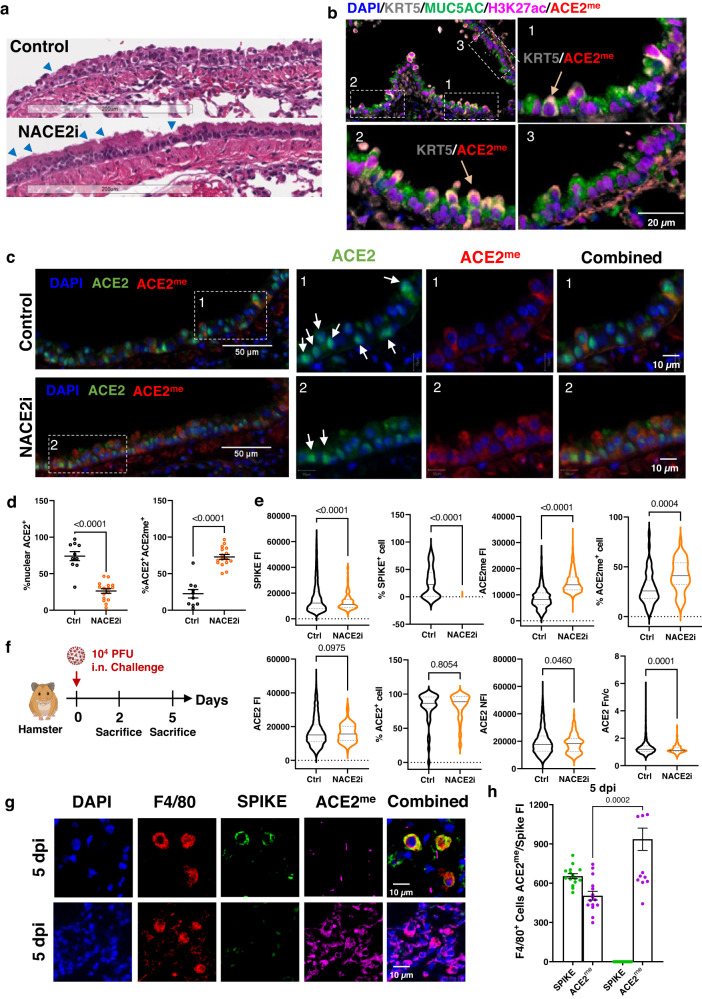
NACE2i induces ACE2 methylation in bronchiolar cells from SARS-CoV-2-infected Syrian golden hamsters. **a** Representative H&E images of SARS-CoV-2-infected golden Syrian hamster lungs treated with either vehicle control (*n* = 7 animals) or NACE2i (*n* = 8 animals). Scale bar, 200 µm. Blue arrows indicate intact ciliated cells. **b** Representative immunofluorescence images of lung section from hamsters (*n* = 3 animals) stained with KRT5 (grey), MUC5AC (green), H3K27ac (magenta), and ACE2^me^ (red) (scale bar 20 µm). Arrows indicate KRT5^+^ cells with ACE2^me^ expression. **c** Representative immunofluorescence images of lung sections from SARS-CoV-2-infected hamsters treated ± NACE2i (*n* = 3 animals/group). Lung tissues were stained for ACE2 (green) and ACE2^me^ (red). DAPI (blue) was used to stain nuclei. Arrow indicated the cells enriched with nuclear ACE2. Scale bars are indicated in white. **d** Bar plots of the population dynamics of nuclear ACE2^+^ and ACE2^+^ACE2^me+^ cells (*n* = 10 FOVs from 3 animals, Ctrl; *n* = 16 FOVs from 3 animals, NACE2i). Data represent mean ± SEM. Two-sided Welch’s *t*-test. **e** Violin plots of the fluorescent intensity of SARS-CoV-2 spike protein, ACE2, and ACE2^me^ in spike^+^ cells and the population dynamics in lung tissues from SARS-CoV-2-infected hamsters treated with either vehicle control (*n* = 44 FOVs from 7 animals) or NACE2i (*n* = 50 FOVs from 8 animals). The ratio of nuclear to cytoplasmic intensity (Fn/c) was calculated for ACE2 (Fn/c > 1 indicates nuclear bias). Black line indicates the medians and dashed line indicates the IQR. Two-sided Welch’s *t*-test. **f** Scheme of the SARS-CoV-2 infection in hamsters. At day 0 post-infection (dpi), each hamster was intranasally (i.n.) inoculated with SARS-CoV-2 (*n* = 3 animals/group), all hamsters were sacrificed and lung tissues were collected at 2 dpi and 5 dpi in a single experiment. **g** Representative immunofluorescence images of lung sections from hamsters infected with SARS-CoV-2 after 5 days (*n* = 3 hamsters/group). Lung tissue was stained for SARS-CoV-2 spike (green), F4/80 (red), and ACE2^me^ (magenta) proteins. DAPI (blue) was used to stain nuclei. **h** Bar plot of the ASI Digital Pathology analysis in (**g**) (3 FOVs/animal from *n* = 3 hamsters/group). Data represent means ± SEM and represent expression of SARS-CoV-2 spike or ACE2^me^ in two groups of F4/80^+^ cells (day 5 SARS-CoV-2 spike positive, day 5 SARS-CoV-2 spike negative). Two-sided Welch’s *t*-test.

Consistent with recent studies^32,33^, there was severe loss of ciliated cells and exposure of underlying basal cells with nuclear rounding in gold Syrian hamsters at 2 dpi (Fig. 5a). However, lung sections from NACE2i-treated hamsters showed increased ciliated cells (Fig. 5a). ACE2^me^ was expressed in exposed KRT5^+^ basal cells but not in MUC5AC^+^ goblet cells (Fig. 5b). By immunohistochemistry, there was nuclear enrichment of ACE2 in control hamsters but not in NACE2i-treated animals, which showed predominantly cytoplasmic and apical surface expression of ACE2, consistent with partly restored ciliation (Supplementary Fig. 6a). Moreover, immunofluorescence staining for ACE2 and ACE2^me^ in control infected hamsters further confirmed that 74.1% of bronchiolar epithelial cells expressed nuclear ACE2 and only a few cells expressed cytoplasmic ACE2^me^ at 2 dpi (Fig. 5c, d), while NACE2i treatment significantly reduced nuclear ACE2 expression and increased the proportion of cells co-expressing ACE2 and ACE2^me^ (Fig. 5c, d).

Treatment with NACE2i significantly reduced or eliminated SARS-CoV-2 spike protein expression and the number of cells positive for SARS-CoV-2 spike protein in the bronchioles of treated hamsters. Conversely, NACE2i significantly upregulated ACE2^me^ in the bronchioles and the proportion of cells positive for ACE2^me^ (Fig. 5e, Supplementary Fig. 6b). Although there was no significant change in global ACE2 and the proportion of ACE2^+^ cells, NACE2i significantly reduced nuclear ACE2 expression, and nuclear to cytoplasmic ratio (Fn/c) analysis showed significantly reduced Fn/c of ACE2, which would be more permissive of ACE2 methylation (Fig. 5e).

Compared with 2 dpi, in situ hybridization to detect SARS-CoV-2 viral RNA only revealed minimal signal in the lungs of infected hamsters at 5 dpi, and the infection was entirely parenchymal^21^. Consistent with our finding in NACE2i-treated hamsters, ACE2 protein was highly enriched in cell nuclei in hamsters at 2 dpi and at the apical border at 5 dpi (Supplementary Fig. 6c). Population dynamics analysis showed significantly increased expression in, but not the proportion of, cells positive for ACE2^me^ at 5 dpi compared with 2 dpi, which was associated with a significant reduction in SARS-CoV-2 spike protein expression (Fig. 5f and Supplementary Fig. 6d–f). Previous studies have shown that monocytes and macrophages can act as latent virus reservoirs to maintain long-term persistence within tissues^34,35^. Consistent with this, we also detected SARS-CoV-2 spike protein expression in F4/80^+^ macrophages in bronchiolar regions at 5 dpi, but macrophages with higher ACE2^me^ expression were negative for SARS-CoV-2 spike protein expression (Fig. 5g, h). These data suggest a possible relationship between ACE2^me^ induction and viral clearance.

### ACE2^me^ is enriched in patient PBMCs following SARS-CoV-2 vaccination and correlates with neutralizing spike antibody titers

**Fig. 6:**
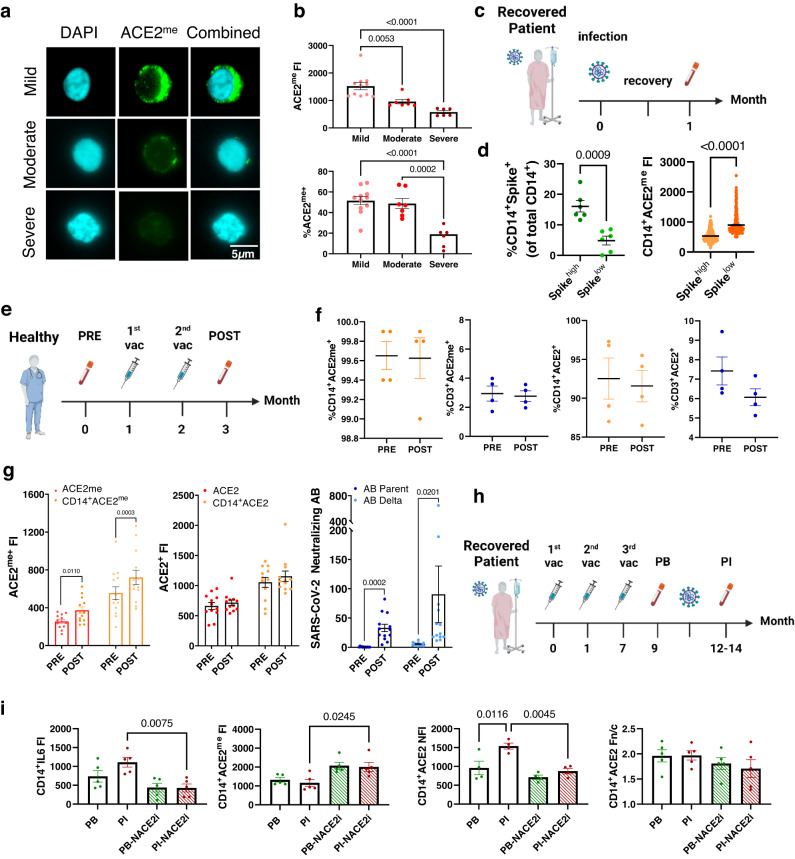
ACE2 methylation is reduced in severe COVID-19 disease, enriched in patient PBMCs following SARS-CoV-2 vaccination, and associated with increased titers of neutralizing SARS-CoV-2 spike antibodies. **a** Representative image (scale bar 5 μm) of PBMCs derived from COVID-19 patients (mild, *n* = 11; moderate, *n* = 7; severe, *n* = 6) using the ASI Digital Pathology system. Cells were permeabilized for immunostaining with ACE2^me^ (green) antibody. DAPI (cyan) was used to visualize nuclei. **b** Dot plot quantification of the fluorescence intensity of ACE2^me^ and population dynamics in mild (*n* = 11 patients), moderate (*n* = 7 patients), or severe COVID-19 patient PBMCs (*n* = 6 patients). Data represent mean ± SEM. One-way ANOVA with Tukey’s post hoc test. **c** Schematic showing blood sample collection from COVID-19 recovered patients (*n* = 12). **d** Depicts the percentage (%) of spike^+^ cells and the fluorescence intensity (FI) of ACE2^me^ from the matched spike positive cohort determined using ASI Digital Pathology analysis of CD14^+^ monocytes of COVID-19 recovered patients (*n* = 12 patients). Data represent mean ± SEM. Samples are group into either spike low expression (spike^low^, *n* = 6 patients) or spike high expression (spike^high^, *n* = 6 patients). Two-sided Welch’s *t*-test. **e** Schematic showing blood sample collection from pre- and post-vaccination individuals (*n* = 13 individuals). **f** FACS analysis of %ACE2^+^ and %ACE2^me+^ cells in CD14^+^ monocytes and CD3^+^ T cells. *n* = 4 individuals. **g** Dot plot quantification of the fluorescence intensity of ACE2^me^ and ACE2 in PBMCs or CD14^+^ monocytes from pre- and post-vaccination individuals. The neutralizing antibody titer for that patient against the parental or delta SARS-CoV-2 strain were also present. *n* = 13 individuals. Data represent mean ± SEM. Two-sided paired *t*-test. **h** Schematic showing blood sample collection from vaccinated patients (post 3^rd^ dose) who subsequently developed and recovered from COVID-19 (*n* = 5 patients). **i** The fluorescence intensity (FI) of IL-6 and ACE2^me^, and the nuclear fluorescence intensity (NFI) of ACE2 in CD14^+^ monocytes from vaccinated patients recovered from COVID-19 treated with or without NACE2i. The ratio of nuclear to cytoplasmic intensity (Fn/c) was also calculated for ACE2. *n* = 5 patients. Data represent mean ± SEM. One-way ANOVA with Tukey’s post hoc test. PB Post-boost, PI Post-infection.

We next examined the dynamics and expression of ACE2^me^ and ACE2 in peripheral blood mononuclear cells (PBMCs) isolated from COVID-19 patients after further optimization and validation of ACE2^me^ immunofluorescence staining in PMBCs (Supplementary Fig. 7a–c). ACE2^me^ expression and the percentage of cells positive for ACE2^me^ were significantly lower in moderate and severe disease compared with mild disease (Fig. 6a, b). Furthermore, SARS-CoV-2 spike protein was expressed in CD14^+^ monocytes in PBMCs isolated from patients who had recovered from COVID-19 (Fig. 6c, d), consistent with recent findings^36^. Similarly, higher levels of SARS-CoV-2 spike protein expression in CD14^+^ monocytes from recovered patients were associated with significantly lower ACE2^me^ expression (Fig. 6d).

We next established whether there was a correlation between the expression of ACE2, ACE2^me^, and neutralizing antibodies targeting SARS-CoV-2 after vaccination. In PBMCs isolated from individuals vaccinated for COVID-19 (Fig. 6e), there was no change in the proportion of ACE2^+^ or ACE2^me+^ cells pre- and post-vaccination, but the expression intensity of ACE2^me^ was significantly higher in PBMCs and CD14^+^ monocytes from vaccinated individuals (Fig. 6f, g and Supplementary Fig. 7f, g). In PBMCs, in 11 out of 13 individuals, ACE2^me^ and neutralizing antibody titers against the ancestral virus were positively correlated, with ten individuals demonstrating increased ACE2^me^ and neutralizing antibody titers and one individual showing only minor changes in both (Supplementary Fig. 7e). The other two individuals demonstrated a decrease in ACE2^me^ and only a moderate increase in neutralizing antibody titers (Supplementary Fig. 7e). An almost identical pattern was observed for antibodies targeting the delta strain of SARS-CoV-2 (Fig. 6g and Supplementary Fig. 7e). In CD14^+^ cells, 12 out of 13 individuals had a positive correlation between increased ACE2^me^ and the neutralizing antibody titers (Supplementary Fig. 7e). Moreover, CD14^+^ cells isolated from donor PBMCs demonstrated much higher expression of ACE2^me^ post vaccination than bulk PBMCs (Fig. 6g). However, there was no change in ACE2 expression in PBMCs and CD14^+^ monocytes post-vaccination (Fig. 6g). Overall, while ACE2^me^ was not expressed in severe disease, post-vaccination individuals and those with mild disease or lower expression of spike protein in monocytes after COVID-19 recovery had PBMCs enriched for ACE2^me^. Therefore, the expression level of ACE2^me^ appears to be associated with reduced disease severity and protection against COVID-19.

Consistent with our findings in the hamster model, ACE2 expression was nuclear in SARS-CoV-2 spike protein-positive monocytes from post-infection (PI) patients, while ACE2 was expressed in the cytoplasm of spike protein-negative cells. NACE2i treatment in vitro reduced nuclear ACE2 expression in infected CD14^+^ monocytes (Supplementary Fig. 7d). We next compared ACE2^me^ and ACE2 expression in PBMCs derived from PI patients and post-booster (PB) vaccination patients (Fig. 6h, i). There were no significant differences in IL6 and ACE2^me^, between PB and PI patients (Fig. 6i). However, nuclear ACE2 expression was significantly higher in monocytes after SARS-CoV-2 virus infection. Again, consistent with the hamster data, in vitro NACE2i treatment significantly decreased IL-6 and nuclear ACE2 expression and induced ACE2^me^ expression in CD14^+^ monocytes. When considered with the pre- and post-vaccination data (Fig. 6e–g), this suggests that there is a virus reservoir in CD14^+^ monocytes/macrophages and that ACE2^me^ expression in CD14^+^ cells is a potential protective signature correlating with neutralizing antibodies targeting SARS-CoV-2 and a reduced reservoir of SARS-CoV-2 spike protein expression.

**Fig. 7:**
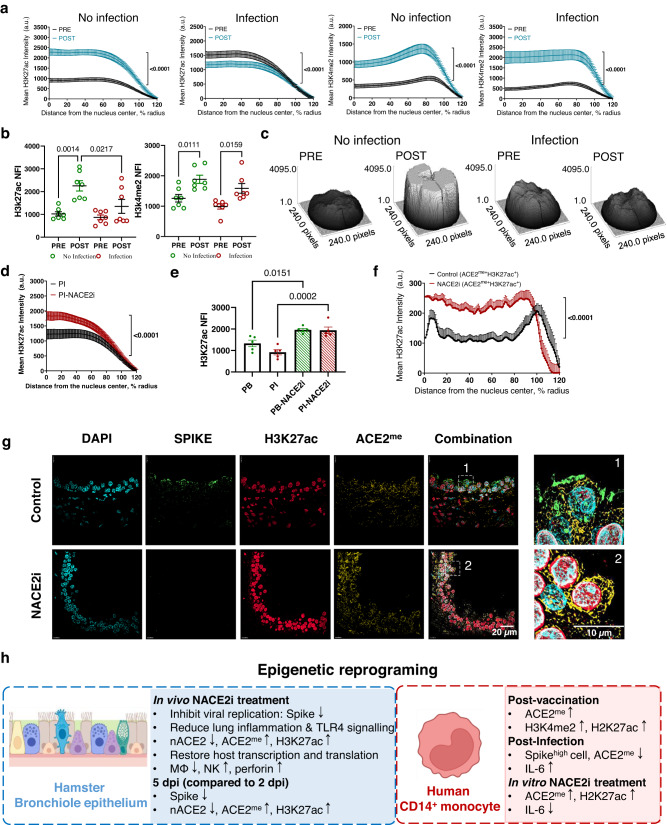
Histone code induction in CD14^+^ monocytes following vaccination or NACE2i treatment. **a** The nuclear distribution of H3K27ac and H3K4me2 were examined in CD14^+^ PBMCs from pre- and post-vaccination donors either remaining uninfected or subsequently becoming infected. Integral radial pixel intensities for H3K27ac (mean ± SEM) are plotted as a function of distance from the nuclear center and clock-scan analysis. *n* = 1 individual (2 FOVs analyzed). Two-sided Kolmogorov–Smirnov test. **b** Dot plot quantification of nuclear fluorescence intensity (NFI) of H3K27ac or H3K4me2 in CD14^+^ PBMCs derived from the samples in (**a**). *n* = 7 individuals. One-way ANOVA with Tukey’s post hoc test. **c** Representative 3D maps of the nuclear density of H3K27ac using the surface plot profile function in ImageJ-Fiji to plot 3D density to demonstrate the nuclear distribution. *n* = 1 individual. **d** Nuclear distribution of H3K27ac was examined in CD14^+^ PBMCs (treated with either control or NACE2i) derived from a post-infection cohort: fully vaccinated and recovered from SARS-CoV-2 infection. Integral radial pixel intensities for each histone mark (mean ± SEM) are plotted as a function of distance from the nuclear center and clock-scan analysis. *n* = 1 individual (2 FOVs analyzed). Two-sided Kolmogorov–Smirnov test. **e** Dot plot quantification of the NFI of H3K27ac in CD14^+^ PBMCs from PB and PI cohorts: fully vaccinated and recovered from SARS-CoV-2 infection and treated with control or NACE2i. *n* = 5 individuals/cohort. One-way ANOVA with Tukey’s post hoc test. **f** Nuclear distribution of H3K27ac examined in ACE2^me+^ cells from lung sections of SARS-CoV-2-infected golden Syrian hamsters treated with vehicle control or NACE2i. Integral radial pixel intensities for each histone mark (mean ± SEM) are plotted as a function of distance from the nuclear center using super-resolution images and clock-scan feature of ImageJ-Fiji. *n* = 5 hamsters/group (1 FOV/animal with ≥ 20 cells analyzed). Two-sided Kolmogorov–Smirnov test. **g** Representative image of FFPE hamster lung bronchioles infected with SARS-CoV-2 and treated with vehicle or NACE2i (*n* = 3 hamsters/group). Super-resolution images are shown. FFPE tissues were stained for ACE2^me^ (yellow), H3K27ac (red), and SARS-CoV-2 spike (green). DAPI (cyan) was used to visualize nuclei. **h** Graphical overview of epigenetic reprograming in hamster bronchiolar epithelium and human CD14^+^ monocytes.

Alterations in histone acetylation and methylation are closely coupled with the priming and reprogramming of regulatory gene signatures in monocytes, suggesting that these aberrant epigenetic marks may contribute to disease onset by initiating or sustaining this pathway. Furthermore, there are recent data showing that epigenetic imprinting with H3K27ac activates promoters and enhancers to induce gene expression in monocytes, particularly the interferon pathway^37^. We therefore profiled H3K27ac in CD14^+^ monocytes pre- and post-vaccination in 14 patients: 7 patients who remained disease free and 7 patients who became infected after vaccination. Pre-vaccination, H3K27ac expression and spatial organization were similar in patients who did or did not develop infection (Fig. 7a–c). However, post vaccination, H3K27ac was significantly induced in individuals who did not become reinfected. The same pattern was also detected in active/accessible nucleosome mark H3.3 (Supplementary Fig. 8a). By comparison, H3K4me2 was globally induced (Fig. 7a, b) but there was no change in the H3K27me3 mark associated with gene repression (Supplementary Fig. 8b). Therefore, the H3K27ac pathway may be an important signature of trained immunity. Targeting nuclear ACE2 with NACE2i successfully induced the H3K7ac mark (Fig. 7d, e) in both PB and PI patients, thereby mimicking the epigenetic tags conducive to trained immunity in individuals where vaccines deliver protection^38^.

We next extended our analysis of the nuclear dynamics of H3K27ac and H3K4me2 using super-resolution and plot profile analyses in individuals pre- and post-vaccination that either did not become infected or who subsequently became infected (Supplementary Fig. 8c, d). Plot profile analysis of H3K27ac in CD14^+^ cells in super-resolution images (Fig. 7a, c) confirmed that H3K27ac expression intensity was much higher in individuals who did not become infected compared with those who did become infected post vaccination (Supplementary Fig. 8c). Moreover, increased H3K27ac fluorescence intensity was associated with areas of reduced DAPI staining – indicative of areas of open chromatin and active transcription - as indicated by plot-profile comparisons of the super-resolution images (Supplementary Fig. 8d).

Finally, we examined induction of H3K27ac in infected hamster lung tissues using both super-resolution imaging and whole slide scanning (Fig. 7f, g and Supplementary Fig. 9a–c). Both super-resolution imaging and digital pathology analysis clearly indicated significant H3K27ac induction along with ACE2^me^ in hamsters treated with NACE2i (Fig. 7f, g and Supplementary Fig. 9b) or those recovered from infection at day 5 (Supplementary Fig. 9a, c). These data suggest that NACE2i boosts or restores host transcription, which may contribute to protection from virus infection, while cellular ACE2^me^ expression can be used as a biomarker of reduced viral replication or efficient protection post vaccination.

## Discussion

Here we show in a pre-clinical hamster model of SARS-CoV-2 infection and human samples the importance of targeting the nuclear ACE2 pathway to treat COVID-19. Targeting nuclear ACE2 with a first-in-class peptide inhibitor significantly improved inflammatory lung pathology, decreased monocyte/macrophage infiltration (a potential viral reservoir), and induced anti-viral NK cells and the cytotoxic molecule perforin. With the application of digital spatial analysis (DSP) to hamsters, we established the gene signatures and pathways hijacked by nuclear ACE2 in response to SARS-CoV-2 infection and targeted by NACE2i, especially those likely to lead to inflammation and prevent viral replication. We also show that targeting nuclear ACE2 induces a protective epigenetic signature, ACE2^me^, in the bronchioles of hamsters treated with NACE2i after SARS-CoV-2 infection and in the monocytes of immunized individuals. This non-histone epigenetic PTM profile could represent trained immunity in the context of COVID-19. Targeting the nuclear ACE2 pathway represents a means to boost vaccine efficacy and ACE2^me^ might be a useful biomarker to monitor protection.

We previously showed that the nuclear ACE2 pathway, fine-tuned by epigenetic enzymes, activates following SARS-CoV-2 infection in human cell lines^4^. This allowed us to develop a highly specific inhibitor to target this axis, NACE2i^4^. Consistent with results in human cell lines, NACE2i inhibited viral infection and reduced or prevented lung damage in NACE2i-treated, SARS-CoV-2-infected hamsters. DSP analysis showed that TLR4 cascades were activated by infection, corresponding to the histopathological inflammation. TLR4 is a transmembrane receptor expressed mainly on immune cells but also at the cell surface of alveolar and bronchial epithelial cells, which plays an important role in initiating inflammatory responses in the lung^39^. A recent in silico study found that SARS-CoV-2 spike protein binds strongly to TLR4^40^, suggesting that SARS-CoV-2 activates TLR4 signaling to mediate inflammation and the consequent lung injury. Also, TLR4 and its signaling are upregulated in patients with severe COVID-19^41^, and S-protein-induced proinflammatory cytokine expression by macrophages and monocytes is TLR4 dependent^42^. Due to elevated proinflammatory IL-6, IL-1β, and TNF-α in severe COVID-19 patients^43^, SARS-CoV-2 may activate TLR4 signaling with a bias towards the proinflammatory MyD88-dependent pathway rather than the anti-viral TRIF-dependent pathway^39^. Interestingly, our DSP analysis of hamster lung tissues showed that the MyD88-independent/TRIF-dependent TLR4 pathway was the top enriched TLR4 cascade after two days of infection. Therefore, recovery from SARS-CoV-2 by hamsters may reflect a balance towards the TRIF-dependent anti-viral response, which provides a first line of protection against SARS-CoV-2 infection rather than triggering a cytokine storm and consequent severe organ injury and possibly death. NACE2i suppressed inflammatory gene expression and lung injury in hamsters, suggesting that NACE2i treatment may protect against SARS-CoV-2-induced hyper-inflammation and lethal organ injury in COVID-19.

DSP allowed us to establish the gene signatures and pathways hijacked by nuclear ACE2 in response to SARS-CoV-2 infection, including complement and DnaJB1 and DnaJB4. Genes upregulated in the lungs of NACE2i-treated hamsters such as *Sftdp* (surfactant protein D) have been shown to play a role in innate immune responses to protect the lung from pathogens, including SARS-CoV-2^44,45^. NACE2i treatment also restored host translational activity by forming a pool of free 40 S subunits and inducing eukaryotic/cap-dependent translation initiation and rRNA processing, which are inhibited by SARS-CoV-2 infection. Moreover, nuclear ACE2 binds to RNA polymerase II (Pol II) upon SARS-CoV-2 infection^4^, and it has been reported that nucleolar Pol II can drive ribosome biogenesis^46^. Hence, increasing 40 S subunit formation by disrupting ACE2 nuclear translocation might suggest that the suppression of Pol II activity promotes ribosome biogenesis. Therefore, we speculate that NACE2i has a dual mode of action: suppressing the lethal inflammation in severe COVID-19 and inhibiting viral replication by restoring host translation of anti-viral cytokines.

ACE2 belongs to a growing number of proteins fine-tuned by PTMs via epigenetic writer and eraser enzymes^1–3^. We previously showed that methylation of ACE2 lysine 31 reduces binding of the spike RBD^4^. Furthermore, demethylation of ACE2 at the RBD by the eraser enzyme LSD1, induced by viral infection, enhanced interactions with the spike protein. Therefore, ACE2 PTMs are critical for mediating host-virus interplay and ultimately the resolution of infection. To explore this further, we developed specific antibodies targeting ACE2^me^ at lysine 31, and our in vivo and in vitro data suggest that ACE2^me^ may be induced as a protective signature of SARS-CoV-2 infection. Analysis of infected bronchiolar epithelial cells demonstrated an inverse correlation between ACE2^me^ expression and SARS-CoV-2 infection. Furthermore, NACE2i induced expression of ACE2^me^ but not global ACE2, with ACE2^me^ only expressed in the cytoplasm and ACE2 enriched in nuclei upon infection. Blocking ACE2 nuclear translocation by NACE2i resulted in accumulation of cytoplasmic ACE2, which may be permissive of ACE2 methylation in the cytoplasm and hence reduced ACE2 translocation to the nucleus (Supplementary Fig. 10). Future studies are now required to identify the methyltransferase involved in ACE2 methylation. Our data suggest that ACE2^me^ might act as an additional layer of protection that can be induced by drugs that target the nuclear ACE2 pathway. Furthermore, our nuclear ACE2 translocation findings support recent evidence that full-length ACE2 is required for SARS-CoV-2 infectivity^47,48^. We have shown that the intracellular, C-terminal domain of ACE2 (the NLS region), present only in cell-bound, full-length ACE2, is essential for the translocation of ACE2 into the nucleus and for viral replication. However, soluble forms of ACE2 naturally present in body fluids do not possess the NLS region so cannot undergo nuclear translocation to support viral replication^47^.

ACE2^me^ expression was significantly repressed in patients with severe infection, while expression of ACE2^me^ increased in vaccinated individuals. This increase in ACE2^me^ correlated with increased antibody titers against SARS-CoV-2 spike protein in most donors. A recent study showed that innate antiviral immunity, particularly mediated by monocytes, is induced in individuals following a second booster dose of the Pfizer vaccine; furthermore, ACE2 is expressed on CD14^+^ monocytes^49–51^. Indeed, we found that this signature was even stronger in CD14^+^ cells of healthy donors post-vaccination than in vaccinated patients who had recovered from COVID-19. However, there was no change in ACE2 expression in PBMCs either post vaccination or in those treated with NACE2i, consistent with the observations in hamster lung tissues. Recent studies have shown that the induction of innate immune memory, or “trained immunity”, is mediated by epigenetic reprogramming^52–54^. Here we demonstrated a potential histone code signature in CD14^+^ monocytes isolated from post-booster vaccinated individuals (Fig. 7h). Our findings suggest that epigenetic re-programming and the distribution of histone mark H3K27ac may provide an additional layer of regulation in determining individuals with prolonged protection versus those at risk of re-infection. This feature of trained immunity, particularly the H3K27ac mark, will need to be validated in future clinical studies. It will also be important to establish if this histone code can differentiate and stratify patients who either fully recover from COVID-19 or develop long COVID. H2K27ac was consistently upregulated in infected hamster bronchiolar cells after NACE2i treatment or on day 5 post-infection, suggesting that host cell transcription activation is important for the inhibition of viral replication or for robust protection against viral infection. Circulating CD14^+^ monocytes appear to express abundant cytoplasmic ACE2^55^. Therefore, we propose that, following COVID-19 vaccination, these monocytes are activated by TLR4 through direct spike protein activation, with induced transcriptional activity upregulating epigenetic enzymes to methylate cytoplasmic ACE2. Further study of H3K27ac-regulated transcription activation is now required.

Viral reservoirs are a key driver of disease progression and persistence in many viral infections, and monocyte/macrophages are one of the major long-lived viral reservoirs in HIV patients following antiretroviral therapy^56–60^. We also detected SARS-CoV-2 spike protein expression in lung macrophages in infected hamsters five days after infection and in circulating monocytes of vaccinated patients who had recovered from COVID-19, indicating that monocytes/macrophages may be a viral reservoir contributing to “long” COVID or “post-acute COVID-19 syndrome (PACS)”^61,62^. This is consistent with a recent study suggesting that SARS-CoV-2 uptake in monocytes and macrophages triggers an inflammatory process that aborts the production of infectious virus but causes systemic inflammation^63^, suggesting that these reservoirs of spike protein could contribute to COVID-19 pathogenesis and also chronic infection in the context of long COVID. Strikingly, monocytes/macrophages with higher expression of ACE2^me^ had significantly lower SARS-CoV-2 spike protein expression, suggesting that ACE2^me^ may act as a protective biomarker of viral persistence in long COVID. Furthermore, long-lived macrophages are the driver of hyperinflammation in convalescent COVID-19 patients^24^. CD163-expressing monocyte-derived macrophages accumulate in the lungs of patients with COVID-19 acute respiratory distress syndrome (ARDS) and are associated with profibrotic responses and pulmonary fibrosis^25^. Our hamster study also showed reduced accumulation of lung macrophages/monocytes and minimal bronchiolar changes in NACE2i-treated hamsters, indicating that targeting nuclear ACE2 is a promising therapeutic strategy to reduce chronic symptoms after acute COVID-19.

Of the two lysine methylation sites in full-length ACE2 (lysine 31 for spike protein binding and lysine 769-771 for IMPα binding), both sites are likely to be important: lysine 31 for virus entry and lysine 769-771 for virus replication. Given that our central focus was to develop a new therapeutic strategy, we opted to block ACE2’s interaction with IMPα at lysine 769-771 by peptide inhibition, since methylating this residue would be technically difficult. At the moment, lysine 31 methylation is best regarded as a biomarker of NACE2i treatment and sustained protection. Further work is needed to establish the mechanisms of regulation governed by the separate methylation events occurring on ACE2 using site-directed mutagenesis.

In conclusion, we show by inhibiting the nuclear ACE2 pathway that therapeutics need to be developed to target not only primary viral replication and inflammation but also to prevent persistent viral reservoir and potential PACS. Future studies will assess and explore the presence of ACE2^me^ imprinting in monocytes from larger cohorts of infected and/or vaccinated individuals developing or not persistent symptoms for “long COVID-19”. Given the unavoidable SARS-CoV-2 variants, the need for continuous booster doses and vaccinated patients suffering from recurrent disease and persistent complications, therapeutics are undoubtedly required in combination with vaccines to enhance and prolong protection.

## Methods

### Fluorescence polarization competition assays

Fluorescence polarization assays were performed using the CLARIOstar Plus plate reader (BMG Labtech, Ortenberg, Germany) with the fluorescein-Ahx-tagged ACE2 peptide sequence ^766^RDRKKKNKARSGEN^779^ manufactured by GeneScript Biotech (Piscataway, NJ), NACE2i peptide sequence MYR-TGIRDRKKKNKARS-OH manufactured by Mimitopes Pty Ltd (Melbourne, Australia), and recombinantly expressed importin-α ΔIBB protein. Each assay contained 50 nM ACE2 FITC, 10 μM importin-α ΔIBB protein, and two-fold serially-diluted NACE2i (starting concentration 400 μM) across 10 wells in a total volume of 200 μL. Fluorescence polarization readings were taken using 96-well black Fluotrac microplates (Greiner Bio-One, Kremsmünster, Austria). Assays were repeated in triplicate and contained a negative control (no inhibitor) and blank (no importin-α ΔIBB protein). Triplicate data were normalized and fitted to a single inhibition curve using GraphPad Prism (GraphPad Software, La Jolla, CA).

### Electrophoretic mobility shift assay

FITC-Ahx-tagged ACE2 peptide (90 μM) was mixed with importin-α ΔIBB protein (100 μM) and NACE2i (500 μM) and electrophoresed through a 1% agarose gel in TB buffer (45 mM boric acid, 45 mM Tris base, pH 8.5) for 90 min at 40 V. ACE2 peptide alone, NACE2i peptide alone, and importin-α ΔIBB alone were used as controls. The gel was first imaged under UV light using a Gel Doc XR+ system (Bio-Rad Laboratories, Hercules, CA) before being stained using Coomassie brilliant blue.

### ACE2 activity assay

Caco2 cells were seeded in T75 flasks using complete DMEM medium as above. Cells were treated with NACEi2 (50 µM) for 24 h. 1 × 10^6^ cells were harvested, and the ACE2 activity assay was performed using the ACE2 Activity Assay Kit (Fluorometric) (ab273297, Abcam, Cambridge, UK) following the manufacturer’s instructions. ACE2 positive control with diluted ACE2 inhibitor was used as a negative control, and cell lysate with lysis buffer only was used as the background control. Fluorescence (Ex/Em = 320/420 nm) was measured in kinetic mode for 30 min (5 min intervals) at room temperature with a Biotek Synergy H4 Multimode Scanner.

### Proximity ligation assay

The Duolink proximity ligation assay was used with PLA probe anti-mouse PLUS (DUO92001), PLA probe anti-rabbit MINUS (DUO92005), and the Duolink In Situ Detection Reagent Red kit (DUO92008) (Sigma-Aldrich). Cells were fixed, permeabilized, and incubated with primary ACE2 antibodies targeting the NLS region and IMPα1 (staining conditions are shown in Supplementary Table 4). Cells were processed according to the manufacturer’s instructions. Finally, coverslips were mounted onto slides and examined as above. Image segmentation and analysis were carried out using QuPath open software (version 0.4.3) for bioimage analysis^64^ with a custom script. An analysis workflow was developed with the following steps: (i) threshold-based cell detection using DAPI staining to create a mask for detected nuclei along with a 5 µm cell expansion setting to denote the cell cytoplasm boundary; and (ii) use of the QuPath subcellular detection module to detect the Duolink-positive spots in the Alexa Fluor 555 channel in each cell. The two functions were wrapped in a script to allow for batch analysis of images and data export. Total dots were counted per cell and then averaged for each cell and experimental group; approximately 100 cells were counted per group.

### NACE2i and ACE2i peptides

For all in vitro studies, NACE2i was reconstituted in water as a 10 mM stock solution and stored at −20 °C until use. Prior to treatment, stock solutions were thawed, prepared to the recommended dose using the appropriate media, and used immediately. For all in vivo studies, NACE2i and Ace2i peptides were reconstituted using the vehicle (0.9% sodium chloride solution) as a stock solution of 10 mg/ml. Formulations were subjected to gentle mixing by inversion and stock solutions stored at −20 °C for up to two weeks. On the day of administration, stock solutions were thawed, prepared to the recommended dose using the vehicle, and injected immediately.

### In vitro monkey plasma assay

Briefly, pooled frozen plasma was thawed at 37 °C prior to experiments. Plasma was centrifuged at 3720 x g for 5 min and any clots were removed. All blanks and working solutions were aliquoted on an Apricot automation workstation (2 μL/well of working solution (100 μM) with 98 μL plasma for NACE2i peptide (CP300638) and control compounds somatostatin acetate and procaine hydrochloride). All reaction plates containing mixtures of NACE2i, somatostatin acetate, and procaine hydrochloride and plasma were incubated at 37 °C in a water bath for 0, 15, 30, 60, 120, 240, and 360 min. Incubation was stopped with 4% H_3_PO_4_ and stop solution (200 ng/mL tolbutamide and 200 ng/mL labetalol in MeOH) to precipitate protein. Each plate was sealed and shaken for 20 min and then centrifuged at 3720 x g at 4 °C for 20 min. After centrifugation, the Apricot workstation was used to transfer 150 μL supernatant to each bioanalysis plate, which was then sealed and shaken for 10 min prior to LC-MS/MS analysis as per the manufacturer’s guidelines. LC: Shimadzu LC 30-AD, MS: QTRAP 6500 + , Autosampler: CTC PAL.

### Mouse tolerance studies

For NACE2i intraperitoneal (i.p.) studies, female C57BL/6 J mice (6-8 weeks, 3 per group) were treated with a single bolus dose of NACE2i at 3, 10, 30, or 100 mg/kg intraperitoneally for seven days. For NACE2i intravenous (i.v.) studies, male C57BL/6 J mice (6–8 weeks, 5 per group) were treated with daily bolus doses of either sodium chloride 0.9% vehicle or NACE2i at 3, 10, or 30 mg/kg intravenously for six days. For ACE2i studies, male C57BL/6 J mice (6 weeks, 5 per group) were treated with daily bolus doses of either saline vehicle or ACE2i at 10, 30, or 100 mg/kg intravenously or at 3, 10, and 30 mg/kg intranasally (i.n.) for six days. All treatments were administered on day 1, and all mice were observed and weighed daily prior to being euthanized for organ examination. All mice were purchased from the Animal Resources Centre (Canning Vale, Western Australia, Australia) and studies were carried out by Agilex Biolabs (St Lucia, Queensland, Australia). Animals were maintained in housing rooms under controlled environmental conditions: individually ventilated cages (IVCs) with a 12/12 light/dark cycle. The ambient temperature is set to 20–25 °C and relative humidity at 30-70% rH. The University of Queensland Animal Ethics Committee assessed and approved all studies (project code 2021/AE000659).

### Hamster tolerance and efficacy studies

Female golden Syrian hamsters (6–8 weeks) were obtained from Janiver Labs (Le Genest-Saint-Isle, France), and studies were conducted by Oncodesign® Biotechnology (Dijon Cedex, France). For tolerance experiments, animals (3 per group) received escalating doses of NACE2i or Ace2i peptide by i.p. injection. Doses were escalated daily (day 1: 25 mg/kg, day 2: 50 mg/kg, day 3: 100 mg/kg) and all animals monitored prior to euthanasia on day 4. Animal viability, behavior, and rectal temperature were recorded every 2 h over a 6-h period post-administration, and body weights were measured daily. For NACE2i efficacy studies, animals (8 per group) were treated with vehicle (i.p. daily on day 0, 1, and 2; sodium chloride 0.9%, Osalia, Paris, France) or NACE2i peptide over a 2-day period i.v. (15 mg/kg, day 0 once and day 1 twice, 8 h apart) or i.p. (100 mg/kg, daily on days 0, 1, and 2). For Ace2i efficacy studies, animals were treated with vehicle or Ace2i peptide (30 mg/kg, i.n., twice daily on days 0 and 1, 8 h apart). For all efficacy studies, peptides were administered to animals 1 h before SARS-CoV-2 infection on day 0 (10^4^ PFU; i.n. administration) with the SARS-CoV-2 strain Slovakia/SK-BMC5/2020 originally provided by the European Virus Archive Global, and all animal were scarified on day 2 post treatment. Animals were maintained in housing rooms under controlled environmental conditions: temperature: 22 ± 2 °C, humidity 55 ± 10%, photoperiod (12 h light/12 h dark), F9 filtered air (CFH), minimum of 12 air exchanges per hour with no recirculation. Each cage is labeled with a specific code. Animal enclosures provide sterile and adequate space with bedding material, food and water, environmental and social enrichment (group housing). All procedures on golden Syrian hamsters were conducted according to French and European Regulations and the National Research Council Guide for the Care and Use of Laboratory Animals. All animal procedures were submitted and approved by the Institutional Animal Care and Use Committee of Oncodesign® Biotechnology, approved by the French authorities, project approval reference D9203202. All hamster tissues provided to the QIMRb were fixed according to approved safety protocols, and only FFPE tissue with no active virus was shipped as per QIMRb COVID-19 safety and animal use project reference P3562.

### Histopathology assessment

Formalin-fixed hamster lung tissues were embedded in paraffin, and sections were stained with hematoxylin and eosin. Slides were read by a veterinary pathologist blinded to the group allocation. Lung histology scores for overall lesion extent, bronchitis, alveolitis, vasculitis, interstitial inflammation, and pneumocyte hyperplasia were assessed using a 0-4/0-5 scale, and scores for each lung were summed to obtain the total histopathological score^19^.

### Viruses

SARS-CoV-2 isolate hCoV-19/Australia/QLD02/2020 (QLD02; GISAID Accession ID EPI_ISL_407896) was initially isolated from nasopharyngeal aspirates from a patient and inoculated into Vero E6 cells. Subsequently, passage 2 of these samples were kindly provided by Queensland Health Forensic and Scientific Services, Queensland Department of Health, and stocks were generated in Vero E6-hTMPRSS2 cells as described in ref. ^65^.

Viral titers were quantified with plaque-forming assays^66^ with some alterations. Briefly, Vero E6 cells were seeded in 12-well plates at a density of 2 × 10^4^ cells per well and left to incubate for 2 days. On the day of infection, viral samples were serially diluted tenfold in serum-free minimum essential medium (MEM; Gibco, Life Technologies, Carlsbad, CA). 250 uL of each sample was incubated with cells for 1 h at 37 °C. Following this, a semi-solid overlay consisting of 4% FCS and 0.1% Low EEO agarose (Sigma-Aldrich, St. Louis, MO) was added. Cells were left to incubate for 2–3 days before fixation with 10% neutral buffered formalin (NBF; POCD Scientific, North Rocks, NSW, Australia) and visualized using crystal violet.

### SARS-CoV-2 infection

In a Physical Containment 2 (PC2) setting, Caco2 cells were seeded at 1 × 10^5^ cells/well using Dulbecco’s modified Eagle medium (DMEM, Gibco) with 10% fetal bovine serum (FBS), 1× L-glutamine (Gibco), and 1× penicillin–streptomycin–neomycin solution (PSN) (Sigma-Aldrich). Cells subjected to siRNA were seeded in DMEM without penicillin-streptomycin.

The next day, cells were treated with NACEi2 (50 µM). siRNA transfection was performed as per the manufacturer’s protocol using Lipofectamine RNAiMAX (Thermo Fisher Scientific) with minor changes. After incubation for 24 h, medium was removed from cells, which were then infected with 200 µL of QLD02 at a multiplicity of infection (MOI) of 1 for 1 h, with consistent rocking every 15 min. After 1 h, virus was removed, and cells were washed with D-PBS three times to remove any remaining viral particles. Inhibitors were freshly diluted and replenished on to cells for another 24 h. Cells subjected to siRNA transfection had their medium replaced with DMEM without penicillin-streptomycin. At 24 hpi, cells and cell supernatants were collected in TRIzol and stored at −80 °C until further processing.

### Virus load determination in lungs by genomic qRT-PCR

For the hamster study, quantification of lung viral load by qRT-PCR was performed using the viral *ORF1ab* gene (Fwd: CCGCAAGGTTCTTCTTCGTAAG, Rvs: TGCTATGTTTAGTGTTCCAGTTTTC, Probe: AAGGATCAGTGCCAAGCTCGTCGCC [5’] Hex [3’] BHQ-1). Viral RNA was extracted using the NucleoSpin® 96 Virus Core Kit (Macherey Nagel, Duren, Germany) and frozen at -80 °C until qRT-PCR. Complete qRT-PCR was run using the SuperScript™ III One-Step qRT-PCR System kit (#1732-020, Life Technologies, Carlsbad, CA) with primers and qRT-PCR conditions targeting the *ORF1ab* gene. Amplifications were performed using a Bio-Rad CFX384™ and its supplied software.

For the Caco2 cell line study, viral titers (PFU equivalents per mL) in the extracted RNA were determined by qRT-PCR using a real-time fluorescent RT-PCR kit for detecting SARS-CoV-2 (two-target) (BGI Genomics, Shenzhen, China) following the manufacturer’s instructions. Positive control (mix of pseudo-virus with target virus genes and internal reference) and blank control (DNase/RNase free water) were used as quality control. The limit of detection was 100 copies/mL. qRT-PCR amplifications were performed using Linegene 9600 and its supplied software. The quantity of viral genomes was calculated by normalizing to a viral stock with a known viral titer.

### Virus TCID_50_ determination in the lungs

Two hours before testing, Vero E6/TMPRSS2 cells were plated in 96-well plates at a density of 25,000 cells per well in a volume of 200 μL of complete growth medium (DMEM with 10% fetal calf serum). Cells were infected with serial dilutions of the day 2 lung homogenate (triplicate) for 1 h at 37 °C. Fresh medium was then added for 72 h. After cell infection, the MTS/PMS assay was performed according to the manufacturer’s protocol (#G5430, Promega, Madison, WI). Briefly, after discarding 100 μL of supernatant, 20 μL of MTS/PMS reagent was added to the remaining 100 μL supernatant. After 4 h, plates were read using an ELISA plate reader and the data recorded.

### Custom antibody generation

Custom polyclonal rabbit antibodies targeting ACE2 sequence QAKTFLD(Kme)FNHEAED were generated by Mimotopes (Mulgrave, Victoria, Australia). Briefly, for antibody generation, a cysteine was incorporated at the C-terminus of the peptide (peptide sequence CQAKTFLD(Kme)FNHEAED) and reacted to conjugate the peptide to an immunogenic carrier protein keyhole limpet hemocyanin (KLH). No special immunization protocols were required to generate anti-methylated peptide antibodies. Rabbits were immunized several weeks apart. The first immunization was with an emulsion of the peptide conjugate with complete Freund’s adjuvant and the second using incomplete Freund’s adjuvant. Potent anti-peptide sera were obtained after several weeks. Methylated/unmodified-peptide antisera were conveniently tested by ELISA, where the sera were titrated on microtiter plates coated with non-methylated peptide and methylated peptide. For methylated peptide antibody enhancement, the non-methylated analog of the peptide used for the immunization was coupled to a gel Sulfo Link (20401:05273’ Thermo Fisher Scientific, Waltham, MA) using the available cysteine residue following the manufacturer’s instructions. The resultant gel was incubated with aliquots of the antisera to absorb antibodies specific to the non-methylated peptide. The resultant antiserum had enhanced specificity for the methylated peptide sequence. To produce affinity-purified antibodies specific to the methylated peptide only, it was necessary to first perform enhancement to remove antibodies from the serum targeting the non-methylated peptide. Specificity of the affinity-purified antibodies was tested by ELISA using both the non-methylated and the methylated peptides coated onto the plate. ELISA results are depicted in Supplementary Fig. 3a,b and show antibody specificity for their targets with appropriate blank/negative controls demonstrating no signals. The ELISA also shows that the antibody specific for the methylated target does not recognize the unmodified peptide and vice versa and is therefore specific for the methylated target. This approach used blanks and negative controls in the ELISA to show that the antibody signals were not background and specific for the target peptide.

### ACE2 immunoprecipitation and immunoblotting assay

Caco2 cells were fixed with formaldehyde (3.7%) and then sonicated with an ultrasonic processor under optimized conditions and lysed with ChIP SDS lysis buffer. ACE2 protein was pulled down using Magna ChIP™ A Chromatin Immunoprecipitation Kit (Sigma-Aldrich) according to the manufacturer’s instructions. Protein elutes were incubated with immunoblot loading buffer containing β-mercaptoethanol at 95 °C. Immunoblot analysis was performed using our custom primary mouse ACE2^me^ antibody or IMPα1 antibody (1:100 dilution), and signals were detected with enhanced chemiluminescence reagents (Western Lightning ECL-Plus; Perkin-Elmer) and the iBright CL1500 Imaging System (Thermo Fisher Scientific). Whole cell extracts were used as a positive control, and a beads-only sample was used as a negative control.

### ACE2 plasmid transfections

Briefly, ACE2_WT or ACE2_K31A mutant sequences were cloned into the pTracer-CMV vector in frame with a C-terminal HA tag. MRC5 cells were transfected with either vector only (VO) plasmid, ACE2_WT, or ACE2_K31A (lysine to alanine mutation at position 31) mutant plasmids using the Lipofectamine 2000 Transfection System Kit according to the manufacturer’s instructions (Thermo Fisher Scientific; 11668019). Cells were then subsequently fixed and stained for ACE2 or ACE2^me4^.

### Immunofluorescence

Immunofluorescence imaging and analysis were carried out using previously established and optimized protocols. Cells were fixed with formaldehyde (3.7%) and then immune-stained with antibodies targeting the SARS-CoV-2 spike protein and custom antibodies targeting ACE2^me^ and ACE2^unmod^. Additional antibodies screened included: CD14, PKC-zeta, HDAC1, NFATc1, Nurr1/Nur77, NFκB-p50, perforin, andc-Rel. Cells were permeabilized by incubating with 0.5% Triton X-100 for 15 min, blocked with 1% bovine serum albumin in PBS, and probed with primary antibodies followed by visualization with secondary donkey anti-rabbit, mouse, or goat antibodies conjugated to Alexa Fluor 488, 568, or 647. Coverslips were mounted on glass microscope slides with ProLong Glass Antifade reagent (Life Technologies).

Protein targets were localized by digital pathology laser scanning microscopy. Single 0.5 μm sections were obtained using ASI Digital Pathology. ASI Digital Pathology characterizes both the fluorescence intensity, similar to normal immunofluorescent imaging protocols, but also provides the ability to count the population of immunopositive or immunonegative cells. This allows the study of population dynamics using custom algorithms and an automated stage. It also allows the imaging and counting of large numbers of cells to achieve statistical power. The microscope uses a 40x or 100x oil immersion lens running ASI software. Final images were obtained by averaging four sequential images of the same section. Digital images were analyzed using automated ASI software (Applied Spectral Imaging, Carlsbad, CA) to determine the distribution and intensities automatically with automatic thresholding and background correction of the average nuclear, cytoplasmic, or total fluorescence intensity, allowing for the specific targeting of expression of proteins of interest. Digital images were also analyzed using ImageJ software (ImageJ, NIH, Bethesda, MD) to determine the total cell fluorescence or cell surface only fluorescence for non-permeabilized cells. Appropriate controls were used for all experiments including primary only control, secondary only control, and ACE2^me^ blocking peptide control. ImageJ with automatic thresholding and manual selection of regions of interest (ROIs) was used to calculate the Pearson’s co-efficient correlation (PCC) for each pair of antibodies. PCC values: −1 = inverse of co-localization, 0 = no co-localization, +1 = perfect co-localization. Tukey’s post hoc test or Welch’s *t*-test was used to determine significant differences between datasets. The Plot-Profile feature of Fiji-ImageJ was used to record the fluorescence intensity of antibody-stained histone PTM targets and DAPI intensity along a line through selected images plotted with the mean ± SEM. ANDOR WD Revolution super-resolution imaging was carried out according to normal specifications for the system, with sample preparation identical to that for immunofluorescence.

### Clock scan analysis

The nuclear distribution of H3K4me2 and H3K27Ac (integral radial pixel intensities) was determined using immunofluorescence imaging, and clock scan analysis was performed^67^. Nuclei were selected and defined with the ImageJ ROI tool for at least 20 cells. All ROIs were then analyzed using the clock scan protocol implemented as a Fiji ImageJ plugin. The intensities were measured separately for the H3K4me2 and H3K27Ac channels. The mean ± SEM of all nuclei were plotted for each % of the radius. The intensity determined outside of the ROI border (at a distance 100–120% of radius) was used for background correction. The Kolmogorov–Smirnov test was used to detect significant differences.

### Immunofluorescent tyramide staining

Opal tyramide staining, unlike traditional immunofluorescence analysis, allows the use of antibodies from the same host species. Imaging and analysis were carried out using established and optimized protocols for permeabilization and antigen retrieval. All FFPE sections were stained using Opal tyramide staining. Samples were dewaxed using a decloaking chamber and prepared using either 0.1% Triton X-100 20 min, Biocare Medical denaturing solution, or Dako pH 6.0/9.0 for antigen retrieval. Sniper+BSA was used for blocking (10 min). Primary antibodies included those targeting ACE2, panCK, F4/80, KRT5, Muc5A, H3K27ac, H3K4me2, CD3, perforin, SARS-CoV-2 spike protein, and custom antibodies targeting ACE2^me^ with VGY or DVG buffers. Primary antibodies were detected with a MACH2 HRP secondary with Opal fluorochromes 520, 570, or 650 and the Opal Tyramide (TSA) 6-Plex Manual Detection Kit (Akoya Biosciences, Marlborough, MA). Final staining conditions are shown in Supplementary Table 2. Imaging and analysis were then carried out as above using the ASI digital pathology platform.

### Hamster lung tissue analysis

Hamster tissues were formalin fixed and paraffin embedded. Tyramide staining was then used with antibodies extensively optimized for concentration, incubation time, antigen retrieval, and pH. Positive controls were employed for each primary antibody target to confirm positive staining on hamster tissue. The ACE2^me^ custom antibody has at least 95% sequence homology with the hamster epitope (one amino acid difference). Final staining conditions are shown in Supplementary Table 2. Biocare Medical Background Sniper (BS966) with 2% BSA was used for blocking, and the primary antibody buffers were either Biocare Da Vinci Green (PD900) or Biocare Van Gogh Yellow (PD902). Primary antibodies were detected with either MACH2 Rabbit HRP (Biocare Medical) for rabbit primary antibodies or mouse HRP (NEF822001EA; PerkinElmer, Waltham, MA) for detection of mouse primary antibodies. Opal PerkinElmer Tyramides (Opal 520, Opal 570, and Opal 650) were used for visualization. The Aperio FL Fluorescence slide scanner was used as a whole slide scanner to image lung FFPE tissue sections to compare staining of custom ACE2^me^ antibody and ACE2 (bulk/commercial antibody). Appropriate controls were used for all experiments including no antibody controls, primary only, or secondary only controls as well as thresholding for positive signals. QuPath analysis software (version 0.4.3)^64^ was used to characterize the expression of ACE2 and ACE2^me^. At least 5 bronchiolar regions were selected per tissue section (with at least 1000 cells counted) for each group (day 2, day 5, and control, i.v. NACE2i, i.p. NACE2i) using QuPath automatic positive cell selection for DAPI. The mean fluorescence intensity was then determined for positive cells for both ACE2 and ACE2^me^ expression. ANDOR WD Revolution super-resolution imaging was carried out according to normal specifications for the system, with sample preparation identical to that for immunofluorescence.

### PBMC staining

Tyramide was used to stain PBMCs fixed with formaldehyde (3.7%) isolated from donor whole blood as per the methods described above and in ref. ^4^ (see Supplementary Table 3 for antibodies).

### Flow cytometry

Fixed PBMCs were washed with PBS and resuspended in PBS containing 1% BSA prior to antibody staining. Primary custom antibodies targeting ACE2^me^ (Mimotopes, Mulgrave, Victoria, Australia), secondary donkey anti-rabbit AF647 (A31573, Thermo Fisher Scientific), ACE2 (sc390851, Santa Cruz Biotechnology, Dallas, TX), CD45 (304027, BioLegend, San Diego, CA), CD3 (562426, BD Biosciences, San Jose, CA), and CD14 (561029, BD Biosciences) antibodies were used. LIVE/DEAD Fixable Near IR (780) viability kit (L34992, Thermo Fisher Scientific) and rabbit IgG isotype control (31235, Thermo Fisher Scientific) was used for flow cytometry staining and acquisition (see Supplementary Table 5 for antibodies). All samples were acquired on an LSR Fortessa cytometer using BD FACSDiva (version 8.01, BD Biosciences, Franklin Lakes, NJ), and data were analyzed using FlowJo **(**version 10.7.1)software. BMC PepTivator stimulation.

PBMCs (5 × 10^5^) were resuspended in 500 μL of RPMI supplemented with L-glutamine (1X), penicillin/streptomycin (1X), and 5% human AB serum. Cells were seeded in a 48-well plate and pre-treated +/- NACE2i (10 μM) for 24 h at 37 °C/5% CO_2_. Following treatment, cells were stimulated with/without PepTivator® SARS-CoV-2 peptide pools (Miltenyi Biotech, Bergisch Gladbach, Germany) covering the sequence domains of the spike glycoprotein of SARS-CoV-2. PBMCs were treated with 20 μL mastermix with equal proportions of PepTivator SARS-CoV-2 Prot_S+ (#130-127-311), Prot_S (#130-126-700), and Prot_S1 (#130-127-041) to ensure complete coverage of the spike protein sequence. Cells were stimulated for 4 h at 37 °C/5% CO_2_ prior to collection for downstream analysis.

### RNA slide preparation for GeoMx digital spatial profiling

FFPE lung tissues from COVID-19-infected hamsters were prepared following the GeoMx digital spatial profiling **(**DSP) slide preparation user manual (MAN-10115-05; NanoString Technologies, Seattle, WA). Briefly, slides were baked in an oven at 60 °C for at least 30 min and then deparaffinized and rehydrated using the Leica Biosystems BOND-RX (Wetzlar, Germany). After epitope retrieval, tissues were treated with proteinase K and then tissue slides were removed from the Leica Biosystems BOND-RX platform followed by in situ hybridization with an RNA probe mix (mouse whole transcriptome atlas and COVID-19 spike-in panel) at 37 °C overnight. After incubation, tissue slides were washed and stained with morphology markers CD45-647 at 1:10 (Novus Biologicals, Littleton, CO; NBP2-34527AF647), PanCK-488 at 1:20 (eBioscience, Thermo Fisher Scientific; 53-9003-82), and DNA dye Syto83 at 1:10 (Thermo Fisher Scientific; S11364) for 1 h at room temperature.

### Region of interest (ROI) selection

Slides were immediately loaded onto the GeoMx-NGS DSP instrument following the GeoMx DSP instrument user manual (MAN-10116-05). 20x high-precision scanning was performed to visualize whole tissue compartments, and regions of interest (ROI) were identified. For each tissue sample, 12 ROIs were selected and assessed by a pathologist from two functional tissue regions: 6 ROIs for bronchiolar tissues and 6 ROIs for alveoli.

### GeoMx DSP NGS library preparation and sequencing

Oligonucleotides from each ROI were released by UV light cleavage and collected into separate wells of 96-well plates. Each oligo sample was uniquely indexed with Illumina’s i5 × i7 dual-indexing system by 18 cycles of PCR followed by two rounds of AMPure XP (Beckman Coulter, Brea, CA; #A63880) purification at a 1.2x bead-to-sample ratio. Libraries were then paired-end sequenced (2 × 27) on an Illumina NovaSeq 6000 with up to 770 million aligned reads in total.

### Data processing and statistical analysis

NovaSeq 6000 FASTQ files for each tissue sample were filtered and demultiplexed using the NanoString DND pipeline (NanoString, version 2.4.0.146) to generate digital count conversion (DCC) files. Sequencing quality control for each sample was performed to ensure > 50% sequencing saturation. For each sample, the LoQ (limit of quantitation) was calculated using the geometric mean of the negative probes multiplied by the geometric standard deviation of negative probes raised to a power of 2. We removed genes below LoQ in all ROIs. The raw counts from the QC-qualified ROIs were then normalized using third quartile (Q3) normalization factors.

Dimensional reduction analysis was performed using the R package *Rtsne* to generate t-distributed stochastic neighbor embedding (t-SNE) clustering. Differential expression analysis was performed by comparing the control and NACEi2-treated tissues within bronchi or alveoli using linear mixed-effects models with the R package *lme4*. Volcano plots were generated with a threshold of fold-change (FC) at 1.5 on a log2 scale and *p*-value 0.05 for a significant cut-off. To identify the biological role of differentially-expressed genes (DEGs), both Gene Set Enrichment Analysis (GSEA) with the R package *fgsea* and Reactome pathway enrichment analysis with the R package *ReactomePA* were performed.

### Cell type deconvolution analysis of NanoString DSP data

Cell type deconvolution analysis was performed using R package *SpatialDecon*^68^. The Q3 normalized count matrix from NanoString DSP data and the safeTME^68^ immune cell profile matrix were used as the query and reference input, respectively, to reveal cell type abundance within each of the selected ROIs. Cell type proportion for each selected ROI was visualized using a stack bar plot, where ROIs were grouped by tissue region (bronchioles vs. alveoli) and experimental condition (control and NACE2i-treated samples). In addition, given the non-normal distribution of the data, the Wilcoxon test was performed to compare each cell type proportion in controls against NACE2i-treated ROIs. The distribution of each cell type and p-value for the comparisons were visualized in box plots.

### IF cell segmentation and measurement

First, the DAPI channel of IF OME-tiff imaging data was used as the input for cell segmentation using the QuPath (version 0.4.3)^64^ cell detection function to generate the cell segments. The defined cell segments were then transferred to ACE2^me^ and SARS-CoV-2 spike channels to extract the single-cell resolution measurements from the respective channels to generate expression intensity matrices (each cell has three measurements). Finally, the expression intensity matrix was log-transformed and standardized (z-score) to minimize the possible effect of outliers.

### Image registration analysis of IF and NanoString DSP data

The Python package SimpleITK^69^ was used for image registration. Both IF images and NanoString images were first downscaled 5x to make the registration more computationally efficient. The DAPI channel in the IF image was cropped and rotated to the same capture area and orientation as the NanoString image and was used as the moving image (query image). NanoString images were converted to grayscale images to transform the pixel data dimension consistent with the IF image and was used as the fixed image (target/reference image). After centralizing the two images, rigid affine transformation was applied for shearing, shifting, and scaling the moving image to align with the fixed image at the same lower resolution as the initial step. Finally, the non-rigid B-spline transformation was applied on affine initialization to refine the local alignment. The mutual information was used as the evaluation matrix to optimize the parameters for both affine and B-spline transformation.

### Integrating IF signals to NanoString DSP-selected ROIs

After registering the IF image with the NanoString image, the optimized transformation matrix could then convert the cells in IF data from the original IF spatial coordinates (*x*, *y*) to newly mapped spatial coordinates (*x’*, *y’*), which were identical to the NanoString spatial coordinates. With this shared coordinate system, ROIs in NanoString data could then be used to select IF cells within the corresponding ROIs by using the transferred spatial coordinates (*x’*,*y’*). The mapping of IF cells to NanoString ROIs provided an additional layer of ACE2^me^, CD3, and SARS-CoV-2 spike expression information onto existing NanoString RNA measurements. Due to the inherent differences between two tissue slices, especially those not immediately adjacent to each other, the ROIs did not always match. In this dataset, ROIs 001 to 005 were manually adjusted after registration, consistent with the morphology (relative to bronchiolar locations and tissue boundaries).

### COVID-19 patients

Whole blood samples were collected from COVID-19 patients (total *n* = 24, mild *n* = 11, moderate *n* = 7, or severe *n* = 6 according to the WHO seven-point ordinal scale) admitted to the Royal Brisbane and Women’s Hospital (RBWH) processed within 4 h of collection. Patients were aged from 29 to 68 years of age. Following venesection, PBMCs were isolated from whole blood using Ficoll-Paque density gradient centrifugation in SepMATE PBMC isolation tubes (STEMCELL Technologies, Vancouver, Canada). Samples were then cytospun onto coverslips at a density of ~100,000 cells per coverslip and fixed with 3.7% formaldehyde. This study was performed in accordance with the NHMRC National Statement on Ethical Conduct in Human Research 2007 (updated 2018). Ethical approval was granted by the Royal Brisbane and Women’s Hospital Ethics Committee (HREC/2020/QRBW/63138) and accepted by the QIMR Berghofer (P3562). All participants provided written informed consent. Samples were stained for ACE2^me^.

### Infected (convalesced) patients

The inclusion criteria for convalescent participants were that they are over 18 years of age, had been clinically diagnosed by PCR with SARS-CoV-2 infection, and had subsequently resolved symptomatic infection and been released from isolation under ethical approval P3618 (QIMR Berghofer Medical Research Institute Human Research Ethics Committee). PBMC were isolated from convalescent participants at a median of 58 days post infection. Patients were aged between 20 and 65 years of age. All participants provided written informed consent. Samples were processed in the same way as for the COVID-19 patient cohort. Samples were stained for SARS-CoV-2 spike protein, ACE2^me^, and CD14.

### Vaccinated patients

PBMC samples were taken from: 1) *n* = 14 participants prior to vaccination (PRE) and four weeks after a second vaccination (POST, Pfizer or Astra-Zeneca), who were further grouped into individuals with (*n* = 7) and without (*n* = 7) subsequent infection; 2) *n* = 5 participants at four weeks after 3^rd^ COVID-19 vaccine (PB, Pfizer or Astra-Zeneca) and four weeks post-infection (PI), following recovery from a positive COVID-19 rapid antigen test. The study was performed under ethical approval P2282 (QIMR Berghofer Medical Research Institute Human Research Ethics Committee). Donors were aged between 34 and 63 years of age. 15% of donors had received the Pfizer vaccination and 85% the AstraZeneca vaccination. All participants provided written informed consent. Samples were processed in the same way as for the COVID-19 patient cohort. The isolated PBMCs were utilized for both immunofluorescent staining and flow cytometry as described above.

### Microneutralization assays

Microneutralization assays were performed as previously with minor modifications^70^. Briefly, serially diluted sera and plasma were incubated with SARS-CoV-2 on Vero cells. Following incubation, cells were fixed, permeabilized, blocked and stained with rabbit anti-NP IgG antibody (Sino Biological, BJ, CN) (1:3000) followed by goat anti-rabbit IgG HRP antibody (Thermo Fisher Scientific) (1:3000). Plates were visualized with ABTS at 405 nm on a SpectraMAX 190. Readings were normalized with cell and virus controls and IC_50_ readings were calculated using GraphPad Prism (La Jolla, CA, version 9.3.1) using the ‘log(inhibitor) vs normalized response’ function.

### Statistics and reproducibility

In all experiments, analyses were performed by applying appropriate statistical tests as detailed in the methods and in the figure legends. The number of biological samples is stated in each figure legend. No statistical method was used to predetermine sample size. All animals were sacrificed at the end of experiments and organs were collected and examined in one go. Two-sided paired/unpaired *t*-tests, the Kolmogorov–Smirnov test, or Welch’s tests were performed for two group comparisons. One-way or two-way ANOVA with Tukey’s post hoc test or mixed-effects model tests were performed for multiple comparisons. No data were excluded from the analyses. Statistical analysis and data plots were performed using GraphPad Prism software (La Jolla, CA, version 9.3.1). All pathology analysis carried out on the hamster model was blinded. All antibodies were extensively optimized and validated prior to use in the experiments.

### Reporting summary

Further information on research design is available in the Nature Portfolio Reporting Summary linked to this article.

## Supplementary information

Supplementary Information

Reporting Summary

## Data Availability

The data generated in this study are provided in this published article and the Supplementary Information file. The RNA sequencing data generated in the NanoString spatial study are available from the Gene Expression Omnibus under accession code GSE233642. Other data generated in this study are provided in the Supplementary Information. Source data are provided with this paper.

## Source data

Source data
