# Supplementary material for: In vivo inhibition of nuclear ACE2 translocation protects against SARS-CoV-2 replication and lung damage through epigenetic imprinting
Source: Nat Commun. 2023 Jun 27;14:3680. doi: 10.1038/s41467-023-39341-4 (PMC10300102; doi:10.1038/s41467-023-39341-4)
Supplement: Supplementary file 1 — Supplementary Information [file 41467_2023_39341_MOESM1_ESM.pdf]

Supplementary Information

In vivo inhibition of nuclear ACE2 translocation protects against SARS-CoV-2 replication and lung damage and through epigenetic imprinting

Wen Juan Tu[1], Michelle Melino[1], Jenny Dunn[1], Robert D. McCuaig[1], Helle Bielefeldt-Ohmann[2,3], Sofiya Tsimbalyuk[4], Jade K. Forwood[4], Taniya Ahuja[1], John Vandermeide[1], Xiao Tan[5], Minh Tran[5], Quan Nguyen[5], Liang Zhang[6], Andy Nam[6], Liuliu Pan[6], Yan Liang[6], Corey Smith[7], Katie Lineburg[7], Tam H. Nguyen[8], Julian D. J. Sng[2], Zhen Wei Marcus Tong[2], Keng Yih Chew[2], Kirsty R. Short[2,9], Roger Le Grand[10], Nabila Seddiki[10] and Sudha Rao[1]

[1] Gene Regulation and Translational Medicine Laboratory, QIMR Berghofer Medical Research Institute, Brisbane, QLD, Australia

[2] School of Chemistry and Molecular Biosciences, The University of Queensland, Brisbane, Queensland, Australia

[3] Australian Infectious Diseases Research Centre, The University of Queensland, Brisbane, Queensland, Australia

[4] School of Biomedical Sciences, Charles Sturt University, Wagga Wagga, NSW, 2678, Australia

[5] Genomics and Machine Learning Lab, Division of Genetics and Genomics, Institute for Molecular Bioscience, University of Queensland, Brisbane, QLD, 4072, Australia

[6] NanoString Technologies Inc., Seattle, WA 98109, USA

[7] Translational and Human Immunology Laboratory, QIMR Berghofer Medical Research Institute, Brisbane, QLD, Australia

[8] Flow and Imaging Facility, QIMR Berghofer Medical Research Institute, Brisbane, QLD, Australia

[9] Australian Infectious Diseases Research Centre, Global Virus Network Centre of Excellence, Brisbane, Queensland, Australia

[10] Université Paris-Saclay, INSERM U1184, CEA, Center for Immunology of Viral, Auto-immune, Hematological and Bacterial diseases (IMVA-HB/IDMIT), Fontenay-aux-Roses, France

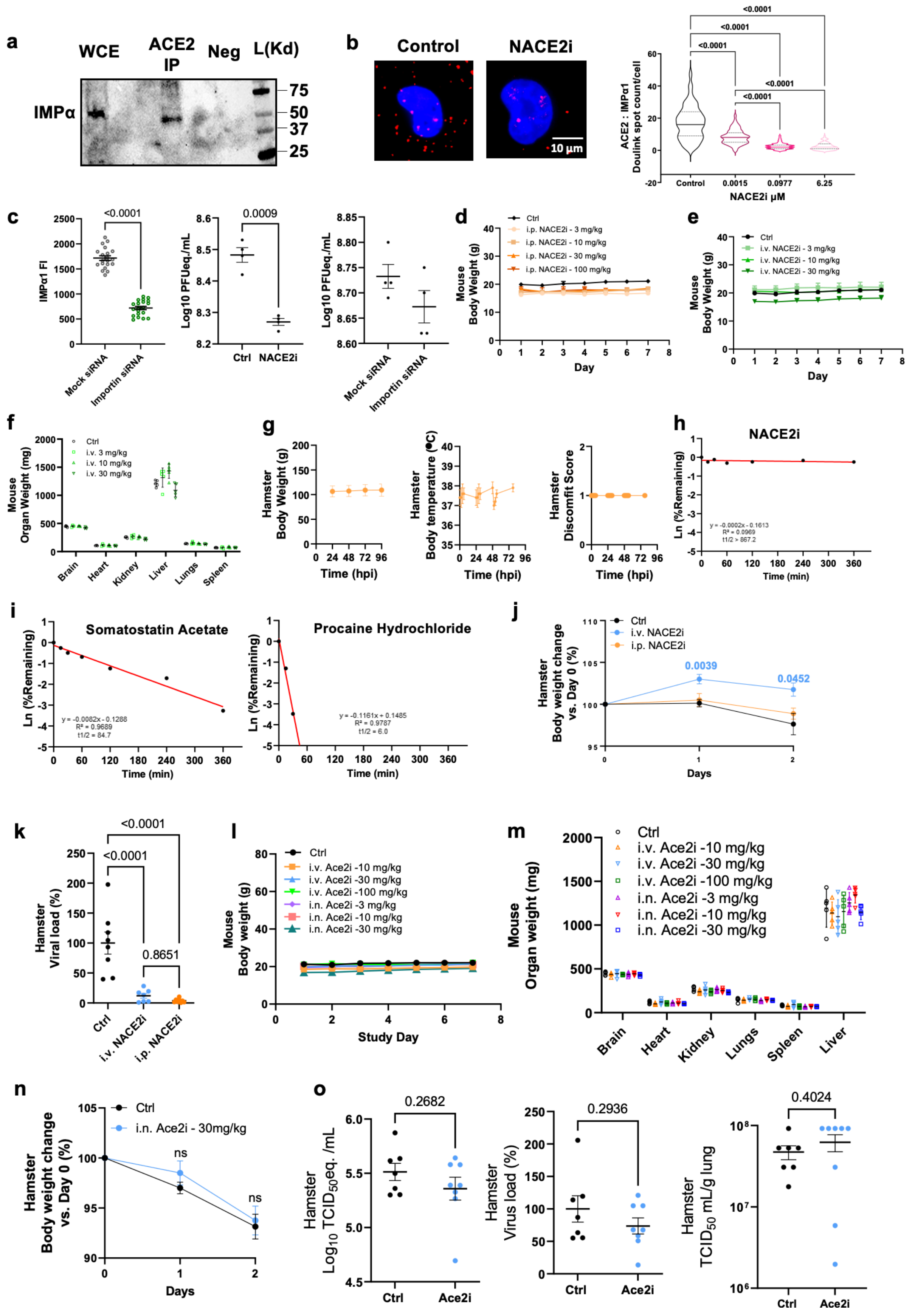

Supplementary Fig 1. NACE2i and ACE2i treatments show no toxicity in animal safety studies.

a. ACE2 was pulled down from Caco2 cells (ACE2 antibody (Abcam, ab15348), which targets the ACE2 NLS region), and samples were then subjected to western blot analysis (n=1) with IMPα antibodies (sc-101292). Lane 1: whole cell extract (WCE), Lane 2: ACE2 pull down, Lane 3: no-antibody control (Neg), Lane 4: precision plus protein ladder (L) used for molecular weight determination (kDa).

b. Duolink® proximity ligation assay measurements of protein interactions for ACE2 (targeting ACE2 NLS region) and IMPα were performed on permeabilized H1299 cells treated with vehicle control or NACE2i (0.0015 μM, 0.0977 μM and 6.25 μM). The Duolink assay produces a single bright dot per interaction within the cell. Representative images (scale bar 10 μm) are shown for vehicle and NACE2i (0.0015 μM). Violin plot of single Duolink spot was shown, n >90 cells counted. One-way ANOVA with Tukey's post test.

c. Caco2 cells were either transfected with siRNA (mock or IMPα) or treated with 50 μM NACE2i for 24 h. Graphs represent IMPα1 FI measured by ImageJ-Fiji. Data represent mean ± SEM, n=20 cells were counted. SARS-CoV-2 RNA expression of N gene was measured by qRT-PCR analysis, and RNA yield is presented as log10 PFU eq/ml. Data represent mean ± SEM, n = 4. Two-sided Welch's *t*-test.

d. Female C57BL/6J mice were treated with a single bolus dose of NACE2i at 3, 10, 30, and 100 mg/kg i.p. Mean body weights (g) from days 1-7 are shown as mean ± SD (n=3/group).

e. Male C57BL/6J mice were treated with daily bolus doses of either saline vehicle or NACE2i at 3, 10, and 30 mg/kg i.v. for six days. Mean body weights (g) from days 1-7 are shown as mean ± SD (n=5/group).

f. Weights of brains, hearts, kidneys, livers, lungs, and spleens collected from male C57BL/6J mice treated with daily bolus doses of either saline vehicle or NACE2i at doses of 3, 10, and 30 mg/kg i.v. for 6 days. Absolute organ weights (mg) are shown as mean ± SD (n=5/group).

g. Female golden Syrian hamsters treated with increasing daily doses of NACE2i 25, 50, and 100 mg/kg by i.p. injection. Animals were randomized on day 1 and treated from day 1 to day 4 (n=3/group). Body weight curves (left), body temperature (middle) and discomfort score (right) are measured and shown as mean ± SD.

h. Stability test of NACE2i in cynomolgus monkey plasma. NACE2i was incubated in plasma for 0, 15, 30, 60, 120, 240, and 360 min. The % remaining of test compound after incubation in plasma was calculated.

i. Stability of somatostatin acetate and procaine hydrochloride in the plasma of *Cynomolgus* monkeys. Test compounds were incubated in plasma for 0, 15, 30, 60, 120, 240, and 360 min. The % remaining of test compound after incubation in plasma was calculated.

j. Body weight curves of female golden Syrian hamsters treated with NACE2i 100 mg/kg by intraperitoneal (i.p.) or 15 mg/kg by intravenous (i.v.) injection (A). Data represent mean ± SEM, n=8/group. Two-way ANOVA with Tukey's post test, *p*-value presented as significant differences compared with vehicle control at each time point.

k. Viral load % is presented relative to control hamster. Data represent mean ± SEM, n=7 i.v. NACE2i; n=8, Ctrl, i.p. NACE2i. One-way ANOVA with Tukey's post test .

l. Male C57BL/6J mice were treated with daily bolus doses of either saline vehicle or ACE2i at doses of 10, 30, and 100 mg/kg via the intravenous (i.v.) route or 3, 10, and 30 mg/kg via the intranasal (i.n.) route for six days. Mean body weights (g) from study days 1-7 are shown as mean ± SD (n=3/group).

m. Organ weights of brains, hearts, kidneys, livers, lungs, and spleens collected from male C57BL/6J mice treated as described above. Absolute organ weights (mg) are shown as mean ± SD.

n. Body weight curves of female golden Syrian hamsters treated with daily bolus doses of either saline vehicle or ACE2i at doses of 30 mg/kg via the intranasal (i.n.) route for two days. Data represent mean ± SEM, n=7, Ctrl; n=8, i.n. NACE2i. Two-way ANOVA with Bonferroni's post test. ns, no significant.

o. qRT-PCR analysis to detect replicates of SARS-CoV-2 RNA in infected lungs from golden Syrian hamsters treated as described above. RNA yield is presented as $\log_{10}$ $TCID_{50}$ eq./mL. Viral load % is presented relative to control and $TCID_{50}$ assay to measure infectious viral titers in infected lungs. Data represent mean ± SEM, n=7, Ctrl; n=8, i.n. NACE2i. Two-sided Welch's t-test .

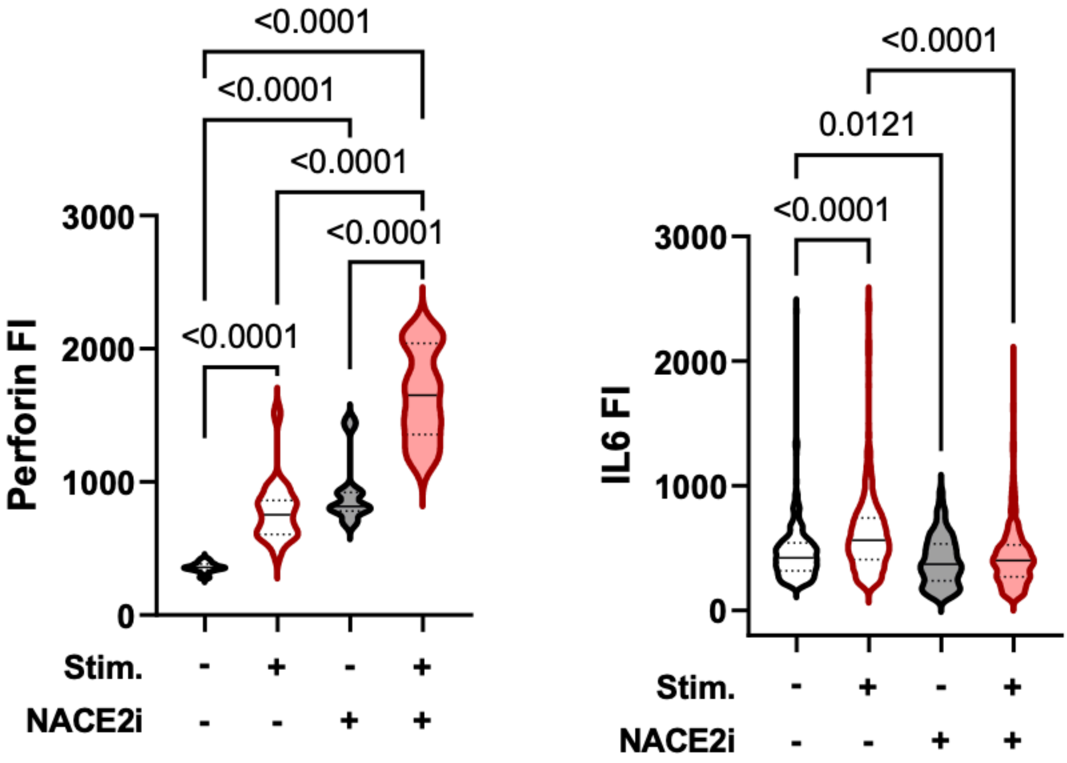

Supplemental Fig 2. NACE2i induces an anti-viral signature and effector signature and abrogates nuclear ACE2.

PBMCs isolated from patient with severe COVID-19 disease were stimulated as described in the methods and treated with vehicle or NACE2i. Samples were stained with IL6 by tyramide staining or perforin by immunofluorescent staining. Cells were scanned using the ASI Digital Pathology microscopy system to determine fluorescent expression. Violin plots represent fluorescent intensity (FI) of perforin (n=1, $\geq$ 20 cells analyzed) and IL6 (n=1, > 300 cells analyzed). Black line indicates the medians and dashed line indicates the interquartile range.. One-way ANOVA with Tukey's post test .

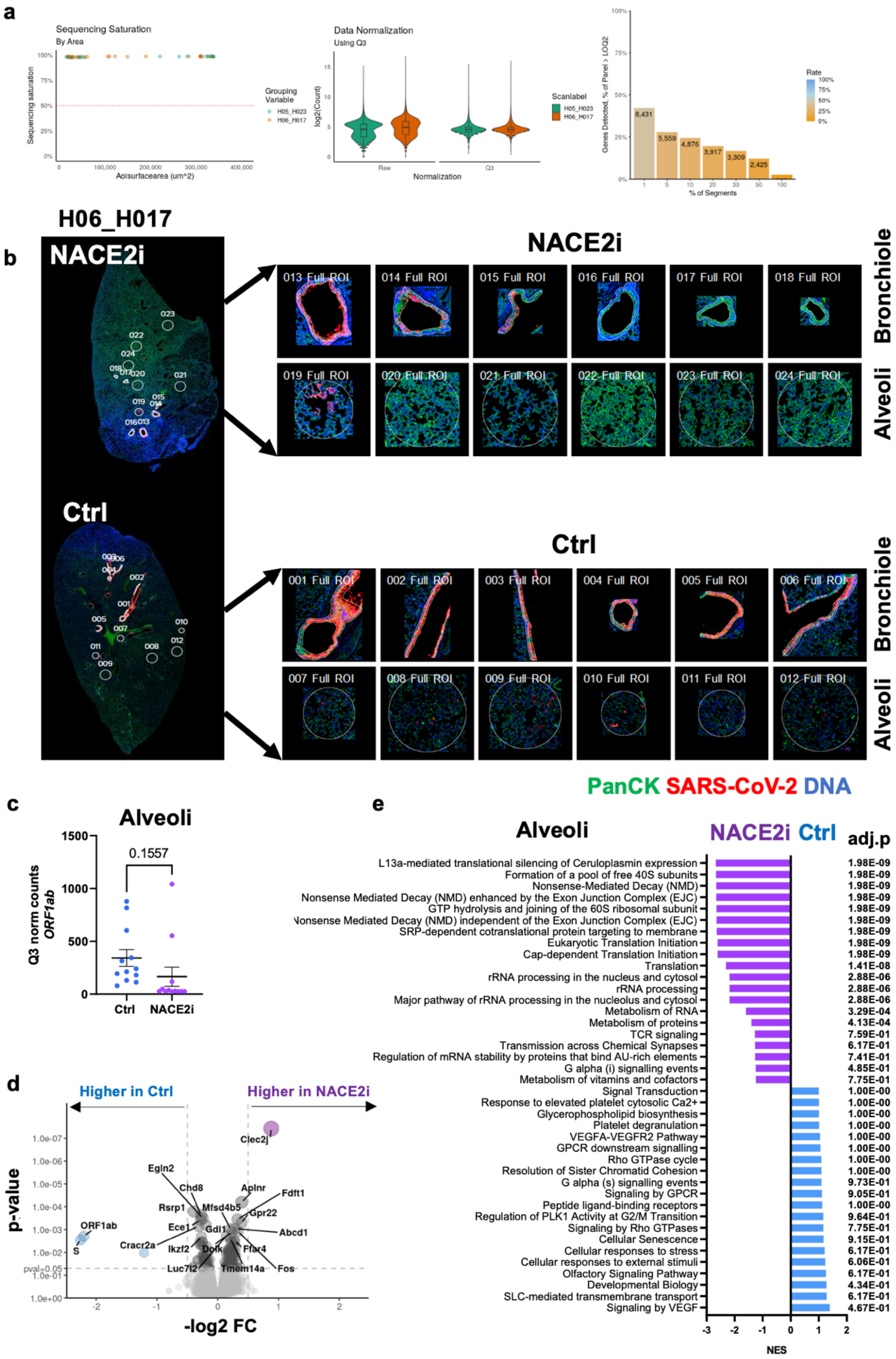

a
Sequencing Saturation
By Area
Sequencing saturation
Aoisurfacearea (um^2)
Grouping Variable
H05_H023
H06_H017
Data Normalization
Using Q3
log2(Count)
Raw
Q3
Normalization
Scanlabel
H05_H023
H06_H017
Genes Detected, % of Panel > LOQ2
% of Segments
Rate
8,431
5,559
4,876
3,917
3,309
2,425
b
H06_H017
NACE2i
Ctrl
NACE2i
013 Full ROI
014 Full ROI
015 Full ROI
016 Full ROI
017 Full ROI
018 Full ROI
019 Full ROI
020 Full ROI
021 Full ROI
022 Full ROI
023 Full ROI
024 Full ROI
Bronchiole
Alveoli
Ctrl
001 Full ROI
002 Full ROI
003 Full ROI
004 Full ROI
005 Full ROI
006 Full ROI
007 Full ROI
008 Full ROI
009 Full ROI
010 Full ROI
011 Full ROI
012 Full ROI
Bronchiole
Alveoli
PanCK SARS-CoV-2 DNA
c
Alveoli
0.1557
Q3 norm counts ORF1ab
Ctrl
NACE2i
d
Higher in Ctrl
Higher in NACE2i
p-value
-log2 FC
pval=0.05
Clec2j
Egln2
Chd8
Aplnr
Fdft1
Rsrp1
Mfsd4b5
Gpr22
ORF1ab
Ece1
Gdl1
Abcd1
S
Cracr2a
Ikzf2
Dohk
Ffar4
Luc7l2
Tmem14a
Fos
e
Alveoli
NACE2i Ctrl
adj.p
L13a-mediated translational silencing of Ceruloplasmin expression 1.98E-09
Formation of a pool of free 40S subunits 1.98E-09
Nonsense-Mediated Decay (NMD) 1.98E-09
Nonsense Mediated Decay (NMD) enhanced by the Exon Junction Complex (EJC) 1.98E-09
GTP hydrolysis and joining of the 60S ribosomal subunit 1.98E-09
Nonsense Mediated Decay (NMD) independent of the Exon Junction Complex (EJC) 1.98E-09
SRP-dependent cotranslational protein targeting to membrane 1.98E-09
Eukaryotic Translation Initiation 1.98E-09
Cap-dependent Translation Initiation 1.98E-09
Translation 1.41E-08
rRNA processing in the nucleus and cytosol 2.88E-06
rRNA processing 2.88E-06
Major pathway of rRNA processing in the nucleolus and cytosol 2.88E-06
Metabolism of RNA 3.29E-04
Metabolism of proteins 4.13E-04
TCR signaling 7.59E-01
Transmission across Chemical Synapses 6.17E-01
Regulation of mRNA stability by proteins that bind AU-rich elements 7.41E-01
G alpha (i) signalling events 4.85E-01
Metabolism of vitamins and cofactors 7.75E-01
Signal Transduction 1.00E-00
Response to elevated platelet cytosolic Ca2+ 1.00E-00
Glycerophospholipid biosynthesis 1.00E-00
Platelet degranulation 1.00E-00
VEGFA-VEGFR2 Pathway 1.00E-00
GPCR downstream signalling 1.00E-00
Rho GTPase cycle 1.00E-00
Resolution of Sister Chromatid Cohesion 1.00E-00
G alpha (s) signalling events 9.73E-01
Signaling by GPCR 9.05E-01
Peptide ligand-binding receptors 1.00E-00
Regulation of PLK1 Activity at G2/M Transition 9.64E-01
Signaling by Rho GTPases 7.75E-01
Cellular Senescence 9.15E-01
Cellular responses to stress 6.17E-01
Cellular responses to external stimuli 6.06E-01
Olfactory Signaling Pathway 6.17E-01
Developmental Biology 4.34E-01
SLC-mediated transmembrane transport 6.17E-01
Signaling by VEGF 4.67E-01
NES

Supplemental Fig 3. GeoMx DSP-NGS analysis in the lung alveoli region of SARS-CoV-2-infected hamsters with and without NACE2i treatment.

a. GeoMx DSP-NGS assay quality control analysis of sequencing saturation (dot plot), data Q3 normalization (violin plot), LoQ2 (limit of quantitation, bar plot). n= 48 ROIs (H05-23: n=24 ROIs, H06-17: n=24 ROIs). Inside the violins, the box plots show medians and interquartile ranges.

b. Selection of regions of interest (ROIs) of the lung in the COVID-19 infected control and i.p. NACE2i-treated hamsters. Left: multi-color immunofluorescent staining with collection of ROIs. Right: ROIs selected from bronchiole (n=6, top) and alveoli (n=6, bottom) regions in each group. PanCK/green, COVID-19/red, DNA Syto 13/blue.

c. Dot plot of Q3 normalized *ORF1ab* gene expression with n=12 ROIs shown in alveoli regions of b) and Fig. 3b. Shown as mean ± SEM. Two-sided Welch's *t*-test.

d. Identification of differentially expressed genes (DEGs) in the lung alveoli regions of COVID-19-infected control and NACE2i-treated hamsters. Horizontal dashed line, p = 0.05; vertical dashed lines, $\log_2$(fold change) = 0.5, fold change. DEGs were defined as p = 0.05 and log2-fold change of 0.5. Top 20 genes are marked and DEGs are colored.

e. Gene Set Enrichment Analysis (GSEA) of top 20 pathways enriched in the lung alveoli regions of COVID-19-infected control and NACE2i-treated hamsters. Adjusted *p*-values are shown on the right.

a

Antibody ID	peptide	Blank	Negative	1:3125	1:6250	1:12500	1:25000	1:50000
ACE2me	Methyl-Lysine 31	0.058	0.056	1.981	1.436	1.041	0.704	0.438
ACE2me	Methyl-Lysine 31	0.059	0.063	1.952	1.415	1.025	0.684	0.414
ACE2me	Unmodified Lysine 31	0.061	0.056	0.305	0.201	0.142	0.076	0.034
ACE2me	Unmodified Lysine 31	0.062	0.057	0.298	0.196	0.134	0.069	0.031
ACE2unmod	Unmodified Lysine 31	0.068	0.065	2.011	1.532	1.102	0.754	0.469
ACE2unmod	Unmodified Lysine 31	0.058	0.056	2.024	1.547	1.117	0.768	0.486

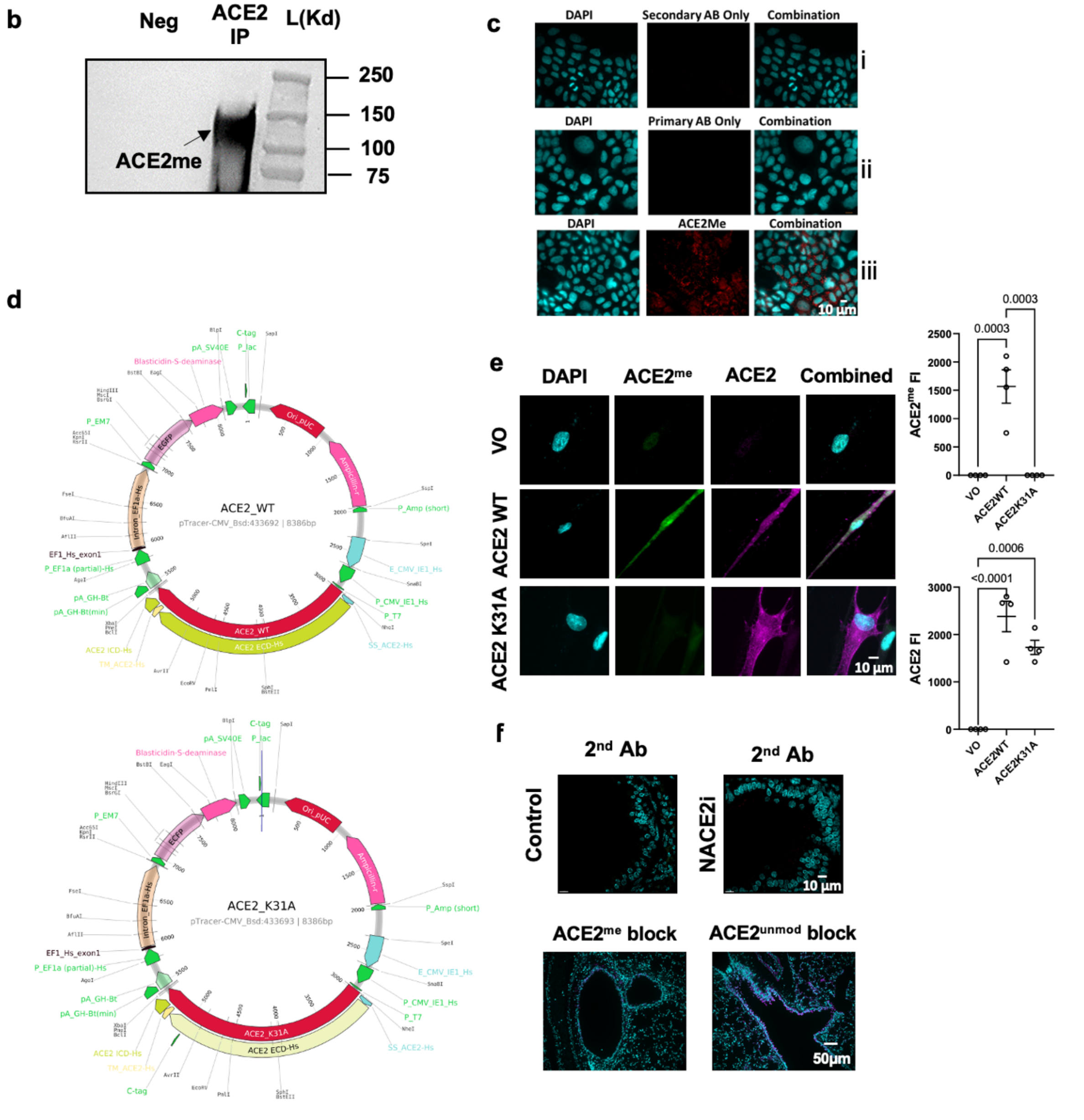

Supplemental Fig 4. The specificity of ACE2me and ACE2.

a. ELISA table showing the specificity of the custom antibodies designed for the ACE2 methylation motif with either no methylation or with the QAKTFLD(Kme)FNHEAED methylation motif. Rabbits 1 and 2 were used to raise antibodies against the modified peptide and also generated the non-methyl antibodies as well via affinity purification. The ELISA data clearly indicate that the antibodies raised against the methylated forms of ACE2 are specific only for the specific methylation and do not recognize or bind to the unmodified peptide (which also works as a negative control for the specificity of the antibodies).

b. ACE2 was pulled down from Caco2 cells (ACE2 antibody (Abcam, ab15348), which targets the ACE2 NLS region), and samples were then subjected to western blot analysis (n=1) with ACE2me antibodies. Lane 1: no-antibody control (Neg), Lane 2: ACE2 pull down, Lane 3: precision plus protein ladder (L) used for molecular weight determination (kDa).

c. Caco2 cells were permeabilized as described in the methods and either stained with secondary antibody anti-rabbit (AF 568) only (i), ACE2^{me} primary rabbit polyclonal antibody only (ii), or ACE2^{me} rabbit polyclonal and anti-rabbit secondary antibody (AF 568) (iii). Stained cells were imaged with the ASI Digital Pathology platform at 100x (n=3). Example images are depicted (scale bar 10 µm).

d. Depicts plasmid maps of ACE2 wild type (ACE2_WT) or ACE2 mutant plasmid (ACE2_K31A: lysine to alanine mutation at position 31).

e. Depicts graphs and example images of FI analysis of MRC5 cells that have been transfected with either ACE2_WT or ACE2_K31A plasmids and stained for either ACE2^{me} or ACE2. Images were taken using the ASI Digital Pathology system (scale bar 10 µm) and data represent mean ± SEM, n = 4. One-way ANOVA with Tukey's post test .

f. Depicted is the control staining for hamster FFPE lung sections from golden Syrian hamsters (n=3). Top (scale bar 10 µm): stained with secondary AB only (no primary antibodies, only secondary AF520, 570 and 650), and bottom (scale bar 50 µm): stained with a cocktail of our custom ACE2^{me} incubated with blocking peptides specific for the methylated ACE2 target sequence prior to staining (ACE2^{me} block: specific for the custom ACE2^{me} antibody (QAKTFLD(Kme)FNHEAED) or blocking peptides not specific for the methylated ACE2 target sequence (ACE2^{unmod} block: unmodified block (QAKTFLDKFNHEAED). Images were taken with the ASI Digital Pathology system, DAPI was used to stain nuclei.

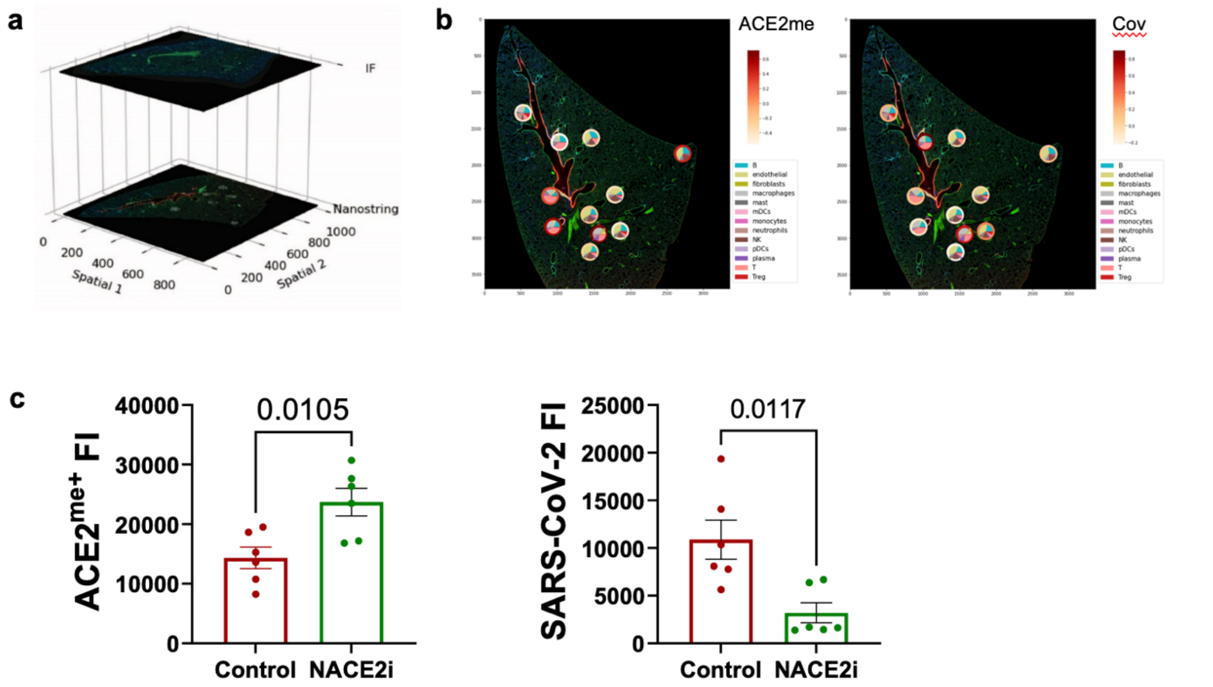

Supplemental Fig 5. IF and NanoString data integration using the image registration method.

a. Affine and b-spline transformation was applied to moving IF images to align with the fixed NanoString image (refer to Methods). 3D plot demonstrates that IF and NanoString data share the same spatial coordinates after image registration, which allows transfer of IF signals to the corresponding ROIs from NanoString data.

b. Spatial visualization of cell type deconvolution using NanoString DSP data (scatter pie plot) and z-score-transformed ACE2^{me} and SARS-COV-2 spike expression from transferred IF data (colored ring) for 12 ROIs in sample H023. Spatial location corresponds to the locations of the ROIs selected from NanoString DSP data. IF image is aligned to NanoString image using image registration as described in the methods to enable direct comparisons of corresponding spatial regions.

c. Bar plots for the ASI Digital Pathology analysis of IF images (n=6 bronchioles). The fluorescence intensity (FI) of SARS-CoV-2 spike and ACE2^{me} are plotted with mean ± SEM. Two-sided Welch's t-test .

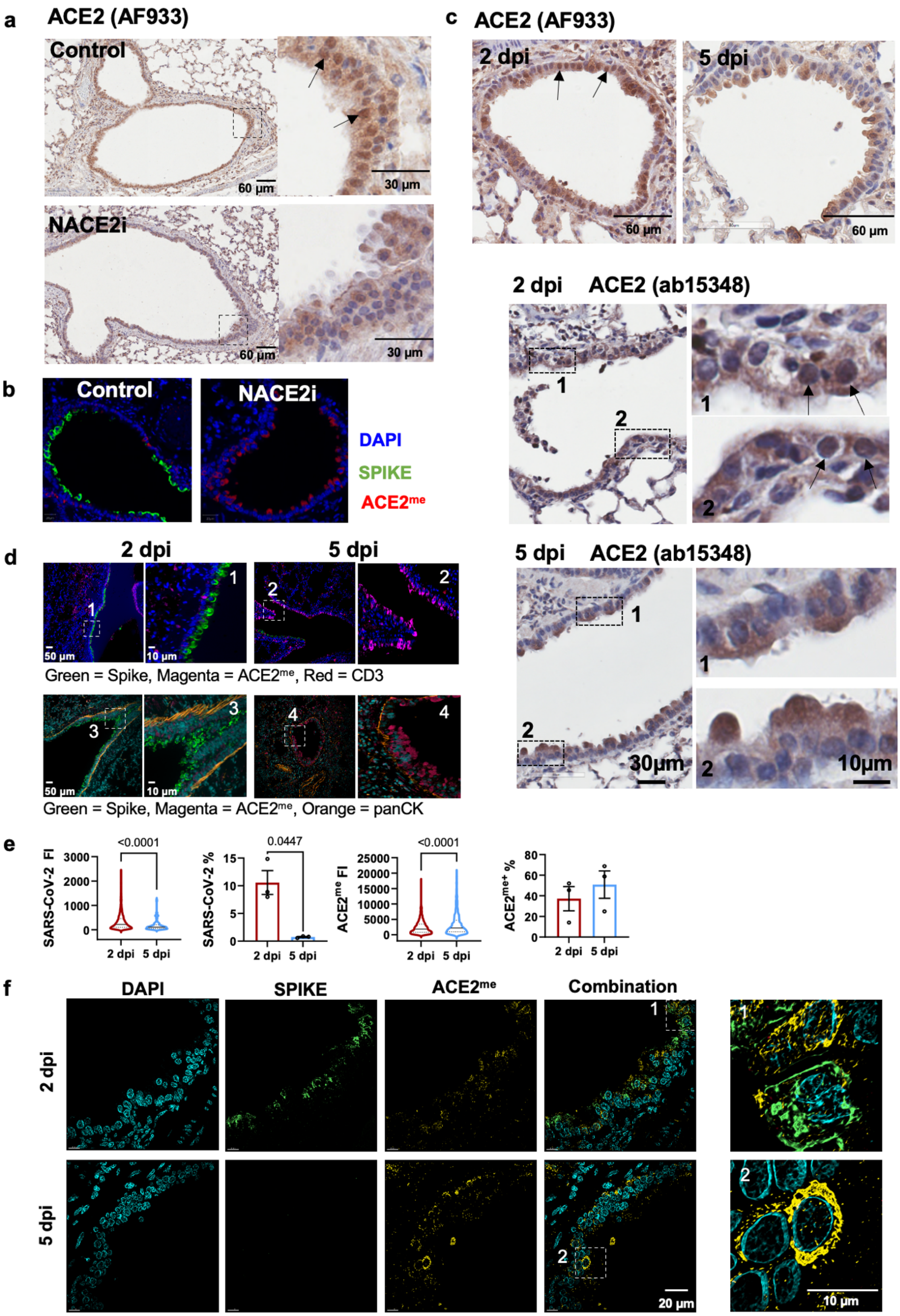

a
ACE2 (AF933)
Control
60 µm
30 µm
NACE2i
60 µm
30 µm
b
Control
NACE2i
DAPI
SPIKE
ACE2me
c
ACE2 (AF933)
2 dpi
60 µm
5 dpi
60 µm
2 dpi ACE2 (ab15348)
5 dpi ACE2 (ab15348)
30µm
10µm
d
2 dpi
5 dpi
50 µm
10 µm
Green = Spike, Magenta = ACE2me, Red = CD3
Green = Spike, Magenta = ACE2me, Orange = panCK
e
SARS-CoV-2 FI
<0.0001
SARS-CoV-2 %
0.0447
ACE2me FI
<0.0001
ACE2me+ %
2 dpi
5 dpi
f
DAPI
SPIKE
ACE2me
Combination
2 dpi
5 dpi
20 µm
10 µm

Supplemental Fig 6. NACE2i induces ACE2 and ACE2me in the bronchial cells of the lung in SARS-CoV-2-infected hamsters.

a. Depicts chromogenic DAB staining of FFPE lung tissue for ACE2 either in control or NACE2i-treated hamsters (n=2). Arrow indicates ACE2 nuclear enriched cells. Scale bar is indicated in black.

b. Representative images of FFPE hamster lung bronchioles of either control or NACE2i-treated hamsters (n=3). FFPE lung tissues were stained with spike (green) and $ACE2^{me}$ (red) and imaged using the ASI Digital Pathology system (scale bar 20 μm). DAPI (blue) was used to visualize nuclei.

c. Depicts chromogenic DAB staining of FFPE lung tissue for ACE2 (R&D AF933 and Abcam ab15348) from golden Syrian hamsters infected with SARS-CoV-2 after 2 or 5 days (n=2). Arrow indicates ACE2 nuclear enriched cells. Scale bar is indicated in black.

d. Depicts example images (taken with the ASI Digital pathology system) of stained FFPE lung sections from golden Syrian hamsters infected with SARS-CoV-2 after 2 or 5 days (n=3). FFPE sections of lung tissue were stained for SARS-CoV-2 spike (green), $ACE2^{me}$ (magenta), CD3 (red) and PanCK (orange); DAPI (blue/cyan) was used to stain nuclei. Scale bars are indicated in white.

e. ASI digital pathology analysis of the fluorescence intensity (bar plots) and population dynamics (violin plots) of SARS-CoV-2 spike and $ACE2^{me}$ (n=3). Bar plots: data represent mean ± SEM. Violin plots: black line indicates the medians and dash line indicates the interquartile range. Two-sided Welch's t-test .

f. Representative images of hamster FFPE lung bronchioles at day 2 (acute infection) or day 5 (recovery) after SARS-CoV-2-infection (n=3) by using the super-resolution Andor WD Revolution Inverted Spinning Disk microscopy system. FFPE lung tissues were stained with $ACE2^{me}$ (yellow) and spike protein (green). DAPI (blue) was used to visualize nuclei. Scale bars are indicated in white.

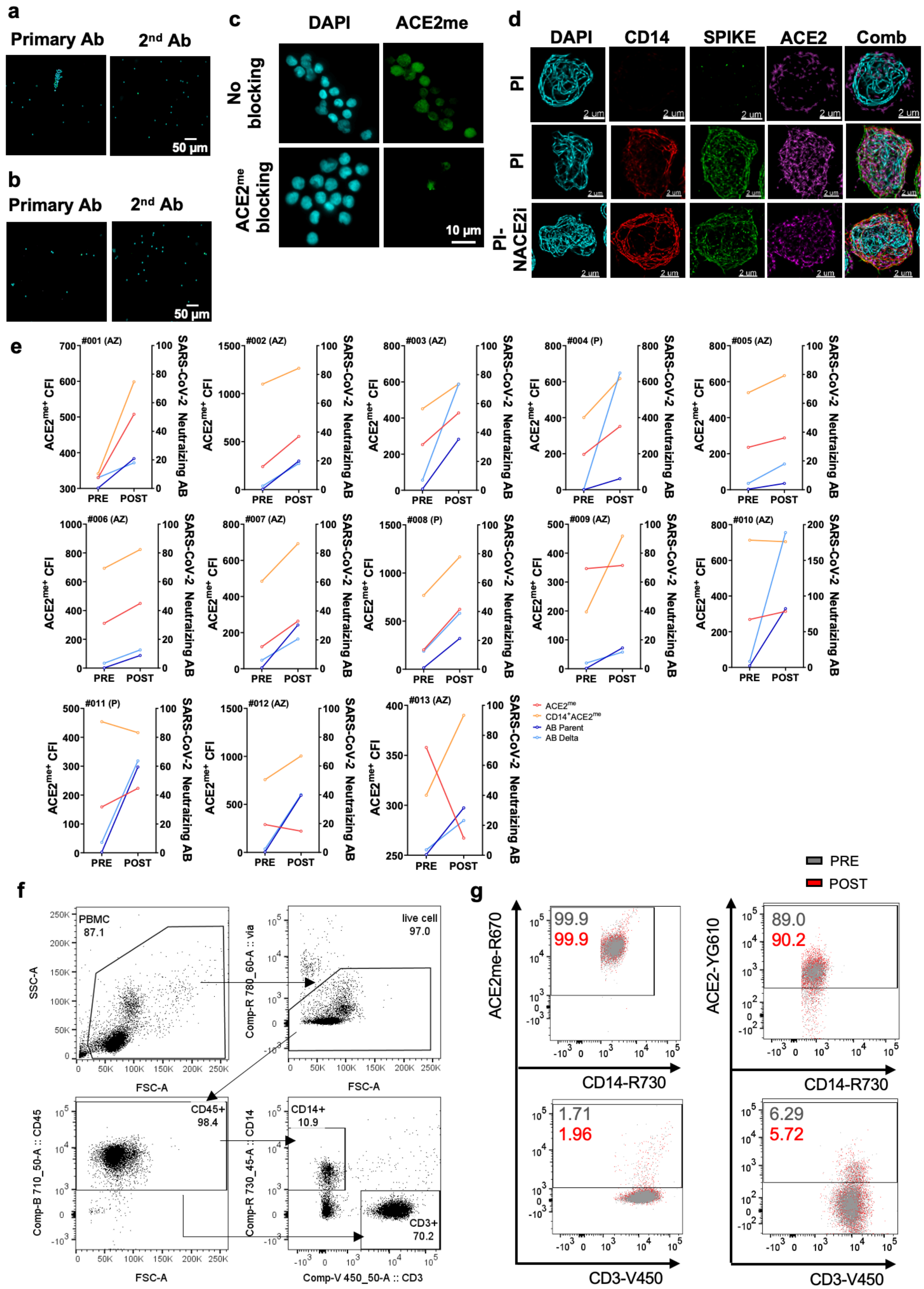
a
Primary Ab
2nd Ab
50 µm
b
Primary Ab
2nd Ab
50 µm
c
DAPI
ACE2me
No blocking
ACE2me blocking
10 µm
d
DAPI
CD14
SPIKE
ACE2
Comb
PI
PI
PI-NACE2i
e
ACE2me+ CFI
SARS-CoV-2 Neutraizing AB
PRE
POST
#001 (AZ)
#002 (AZ)
#003 (AZ)
#004 (P)
#005 (AZ)
#006 (AZ)
#007 (AZ)
#008 (P)
#009 (AZ)
#010 (AZ)
#011 (P)
#012 (AZ)
#013 (AZ)
ACE2me
CD14+ACE2me
AB Parent
AB Delta
f
PBMC
87.1
SSC-A
FSC-A
live cell
97.0
Comp-R 780_60-A :: via
CD45+
98.4
Comp-B 710_50-A :: CD45
CD14+
10.9
CD3+
70.2
Comp-R 730_45-A :: CD14
Comp-V 450_50-A :: CD3
g
PRE
POST
ACE2me-R670
CD14-R730
99.9
99.9
ACE2-YG610
89.0
90.2
CD3-V450
1.71
1.96
6.29
5.72

Supplemental Fig. 7. ACE2^{me} is inhibited in severe COVID-19 disease and enriched in patient PBMCs following SARS-CoV-2 vaccination.

a. Representative images (scale bar 50 μm) of PBMCs (n=3) taken with the ASI Digital pathology platform. Cells were stained with either primary antibody only (ACE2^{me}) or secondary antibody only (anti-rabbit AF568). DAPI (cyan) was used to visualize nuclei.

b. Representative images (scale bar 50 μm) of PBMCs (n=3) taken with the ASI Digital pathology platform. Cells were stained with either primary antibody only (ACE2) or secondary antibody only (anti-goat AF647). DAPI (cyan) was used to visualize nuclei.

c. Representative ASI Digital pathology images (scale bar 10 μm) of PBMCs (n=3) treated with or without blocking peptides specific for the target sequence of custom ACE2^{me} antibody (QAKTFLD(Kme)FNHEAED) prior to the staining of ACE2^{me}. DAPI (cyan) was used to visualize nuclei.

d. Representative image (scale bar 2 μm) of CD14^{+} PBMCs derived from from vaccinated patients recovered from COVID-19 with or without NACE2i treatment (n=3). Images were taken using the super-resolution Andor WD Revolution Inverted Spinning Disk microscopy system. Cells stained with CD14 (red), SARS-CoV-2 spike (green), and ACE2 (magenta). DAPI (cyan) was used to visualize nuclei.

e. Plots of individuals pre- and post-vaccination showing the population dynamics of ACE2^{me+} cells compared with the neutralizing antibody titer for that patient against the parental or delta SARS-CoV-2 strain. n=13 individuals.

f. Representative gating strategy of flow cytometry for Figure 6f. CD14^{+} monocytes were identified by live CD45^{+}CD14^{+}. CD3^{+} T cells were identified by live CD45^{+}CD3^{+}. Cells expressing ACE2^{me} and ACE2 are shown in Supplementary Figure 7g.

g. Representative flow cytometry dot plots of ACE2^{me} and ACE2 expression by CD14^{+} monocytes and CD3^{+} T cells from pre- and post-vaccination individuals. n=4 individuals.

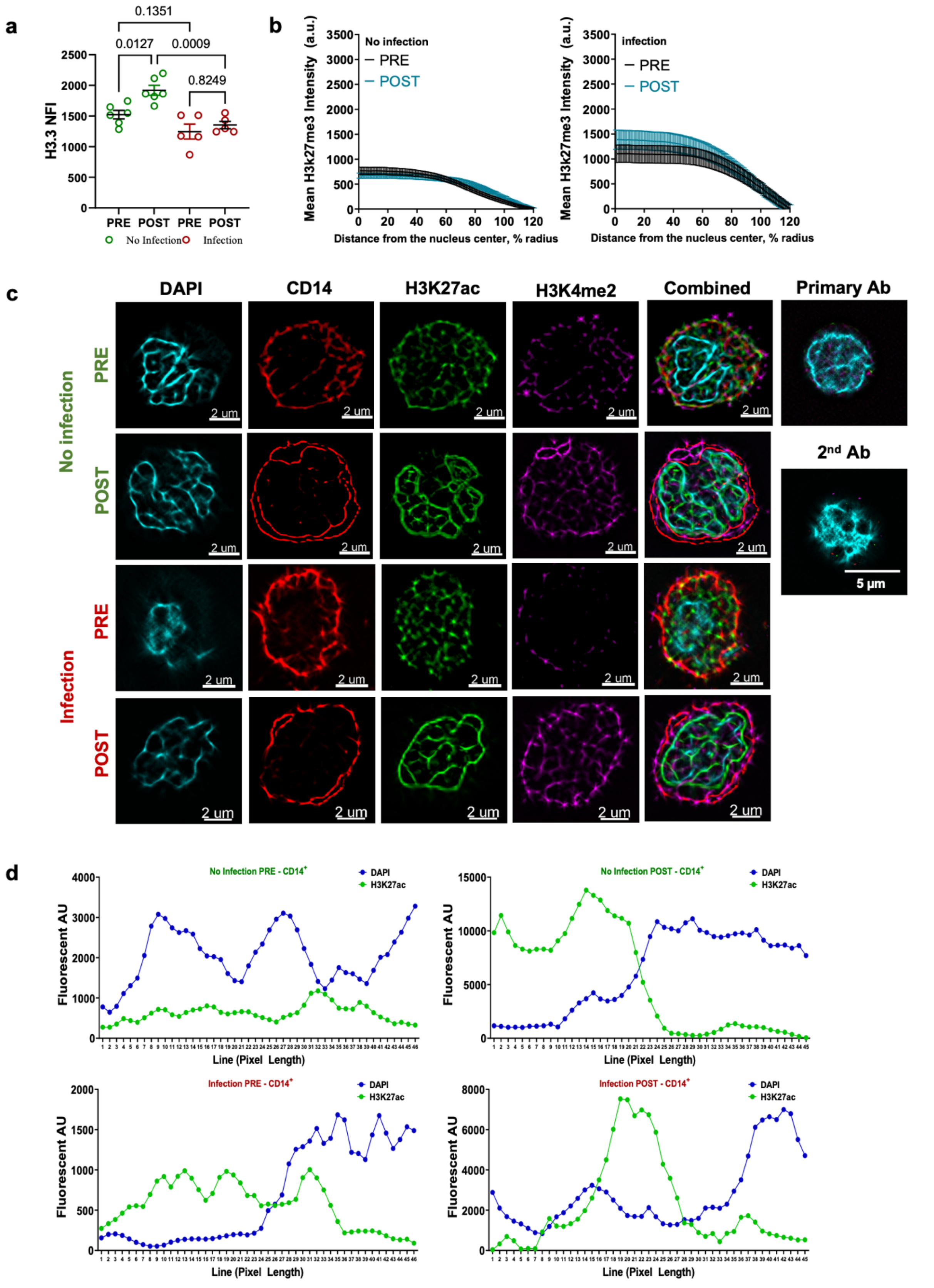

Supplemental Fig. 8. Histone mark expression in CD14^{+} cells from pre/post vaccination patients.

a Dot plot quantification of the nuclear fluorescence intensity (NFI) of H3.3 in $CD14^+$ PBMCs derived from pre- and post-vaccination donors either remaining uninfected or subsequently becoming infected. Data represent mean ± SEM, n=6 individuals per cohort. One-way ANOVA with Tukey's post test .

b The nuclear distribution of H3K27me3 was examined in $CD14^+$ PBMCs derived from pre- and post-vaccination donors either remaining uninfected or subsequently becoming infected. Integral radial pixel intensities for H3K27me3 (mean ± SEM) are plotted as a function of distance from the nuclear center using the ASI Digital Pathology system and clock-scan analysis (n=20 cells analyzed). .

c The Andor WD spinning disc super resolution system was used to examine the nuclear distribution of H3K27ac and H3K4me2 in $CD14^+$ PBMCs derived from pre- or post-vaccination either remaining uninfected or subsequently becoming infected (n=3). Cells stained with either Primary Ab only or Secondary Ab only were examined as negative control by the same system. Scale bar is 5 µm. DAPI (cyan) was used to visualize nuclei.

d Plot profile analysis was carried out to understand the effect of vaccination on H3K27ac or H3K4me2 in relation to the DAPI stain (high intensity stain indicates closed chromatin, whereas reduced intensity stain indicates more open chromatin based on density of DAPI).

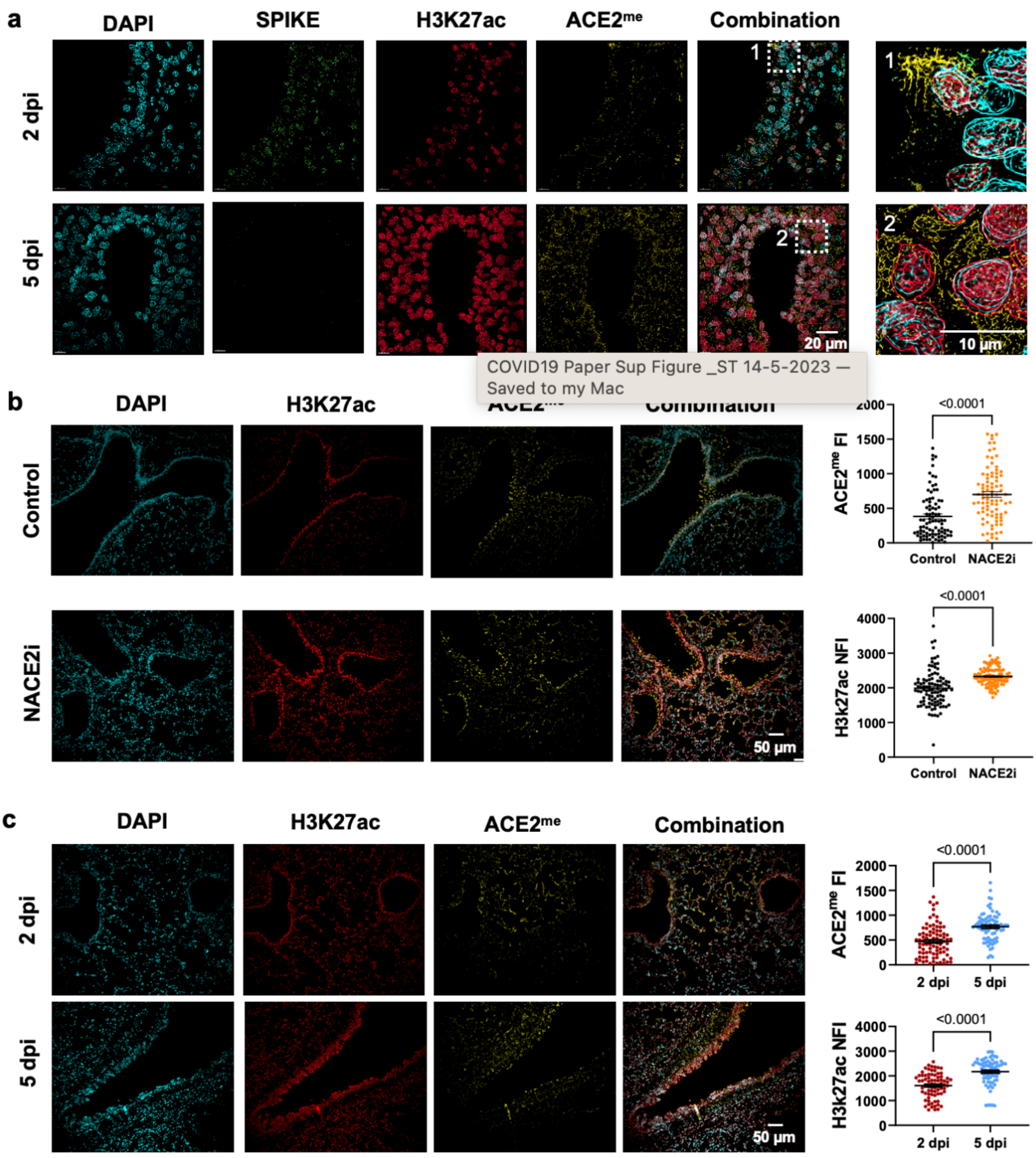

Supplemental Fig. 9. Histone expression in the bronchial cells of SARS-CoV-2 infected hamsters.

a. Representative image of hamster FFPE lung bronchioles either at day 2 (acute infection) or day 5 (recovery) after SARS-CoV-2-infection (n=3). Samples imaged using the super-resolution Andor WD Revolution Inverted Spinning Disk microscopy system. FFPE tissues were stained for ACE2^{me} (yellow), H3K27ac (red), and spike (green). DAPI (cyan) was used to visualize nuclei. Scale bars are indicated in white.

b. Depicts example images (taken with the ASI digital pathology system, scale bar 50 µm) of FFPE lung bronchiole sections from Golden Syrian hamsters treated with either vehicle or NACEi2. Lung sections were stained with H3K27ac (red) and ACE2^{me} (yellow). DAPI (cyan) was used to visualize nuclei. Dot plot quantifications of fluorescence intensity (FI) of ACE2^{me} (n=88 cells analyzed) and nuclear fluorescence intensity (NFI) of H3K27ac (n=100 cells analyzed) are presented as means ± SEM. Two-sided Welch's *t*-test.

c. Depicts example images (taken with the ASI digital pathology system, scale bar 50 µm) of FFPE lung bronchiole sections from Golden Syrian hamsters either at day 2 (acute infection) or day 5 (recovery) after SARS-CoV-2-infection. Lung sections were stained with H3K27ac (red) and ACE2^{me} (yellow). DAPI (cyan) was used to visualize nuclei. Dot plot quantifications of fluorescence intensity (FI) of ACE2^{me} (n=88 cells analyzed) and nuclear fluorescence intensity (NFI) of H3K27ac (n=75 cells analyzed) are presented as means ± SEM. Two-sided Welch's *t*-test .

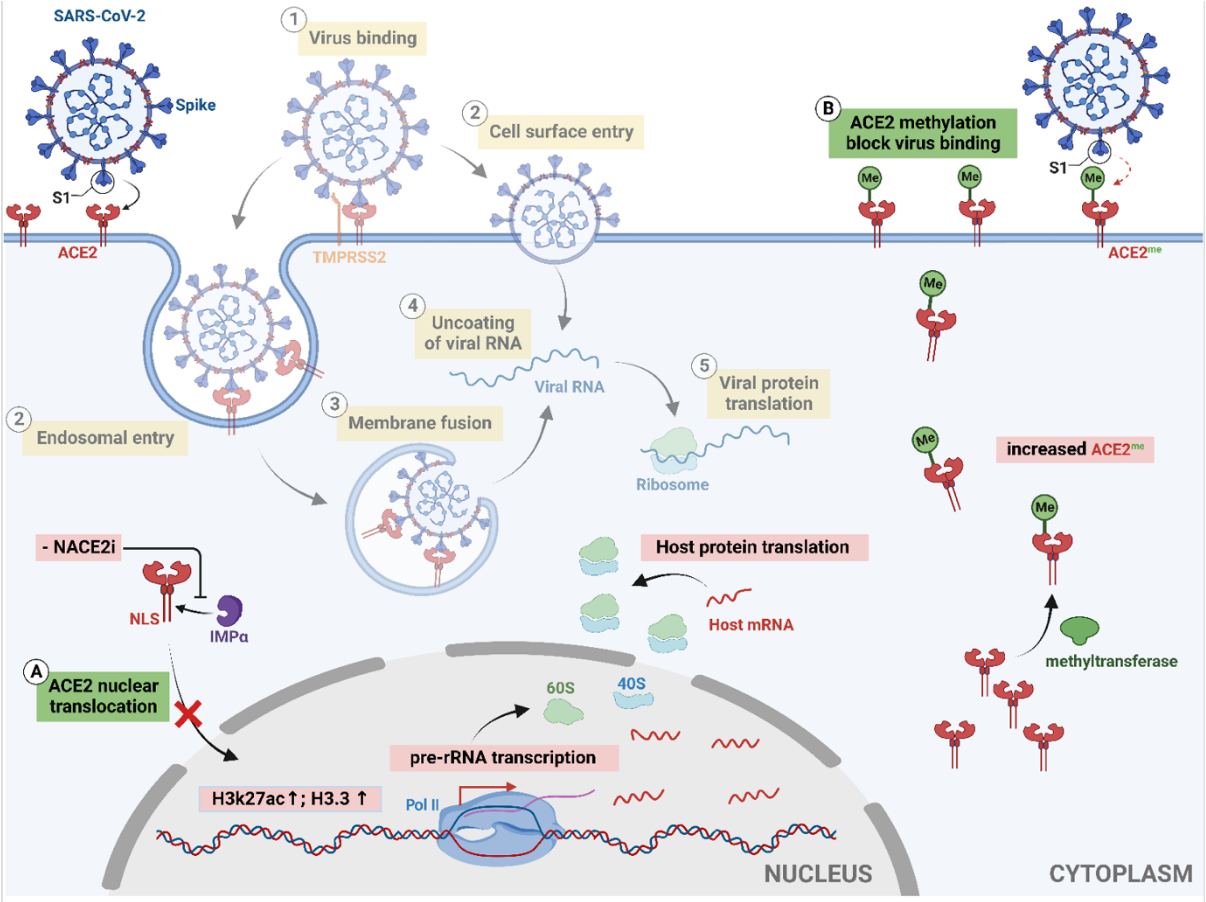

Supplemental Fig. 10. Overview of SARS-CoV-2 entry and replication in host cells and its inhibition by the NACE2i epi-drug.

SARS-CoV-2 virus enter the host cells via spike (S1)/ACE2 binding ①-③ and hijacks host ribosomes to inhibit host mRNA translation ④-⑤.

There are two lysine methylation sites in full-length ACE2: Lys^{31} for spike protein binding and $Lys^{769\text{-}771}$ for IMPα binding. Our NACE2i epi-drug reduces virus replication by (A) blocking IMPα/ACE2 nuclear localization signal (NLS) binding to reduce nACE2 (nuclear ACE2), thereby increasing $ACE2^{me}$ at lysine 31 (accumulated cytoplasmic ACE2 methylated at lysine 31 by methyltransferase), restoring host mRNA transcription (upregulated active transcription marks of H2K27ac and H3.3), restoring host translation (promoting pre-rRNA transcription), and consequently enhancing antiviral responses (increased perforin expression); and (B) SARS-CoV-2 glutamine 493 in the receptor-binding domain (RBD) binds to ACE2 lysine 31 for cell entry. Therefore, increased $ACE2^{me}$ at lysine 31 reduces spike/ACE2 binding affinity, blocking virus entry.

Supplementary Table 1. Overview of the clinical features observed in animal safety studies. C57BL/6 mice were treated with a single intraperitoneal (i.p.) bolus dose or daily intravenous (i.v.) doses of NACE2i for 6 days. All mice were monitored for abnormalities for the duration of the study.

	NACE2i via IP Route				*Vehicle*	*NACE2i via IV Route*		
**NACE2i dose (mg/kg)**	**3**	**10**	**30**	**100**	**0**	**3**	**10**	**30**
n	*3*	*3*	*3*	*3*	*5*	*5*	*5*	*5*
No abnormalities:	3	3	0	0	5	5	5	0
Reduced Activity								
- Mild	0	0	3	2	0	0	0	4
- Moderate	0	0	0	2	0	0	0	5
Palpebral Closure								
- Mild	0	0	1	0	0	0	0	1
Piloerection								
- Mild	0	0	0	2	0	0	0	0
Diarrhoea								
-Mild	0	0	0	1			NA	
Right limb raised;								
-Mild	0	0	3	0			NA	
Hunched Posture								
- Mild		NA			0	0	0	1
Tremors								
- Mild		NA			0	0	0	5

Supplementary Table 2. Tissue antibody staining conditions (hamster).

Antibody	Antigen Ret.	Blocking	Primary	Secondary	TSA
**ACE2^me^** Rao custom antibody	No AR	Sniper + BSA 10 min	1:500 DVG 60 min	MACH2 Rabbit HRP 45 min	Opal 650 15 min
**ACE-2** R&D Systems (AF-933)	No AR	Sniper + BSA 10 min	1:500 DVG 60 min	MACH2 Rabbit HRP 20 min	Opal 650 15 min
**ACE2** Abcam (ab15348)	Dako pH 9.0 25'@95°C	Hydrogen peroxide + TBS 10 min	1:1000 DVG 60 min	MACH2 Rabbit HRP 30 min	HRP 5 min
**CD3** Dako (A0452)	Dako pH 9.0 20'@100°C	Sniper + BSA 10 min	1:3000 VGY 30 min	MACH2 Rabbit HRP 15 min	Opal 650 10 min
**F4/80** Abcam (ab6640)	Diva 20'@100°C	Sniper + BSA 10 min	1:4000 VGY 20 min	BC Rat probe and polymer 10+10 min	Opal 570 10 min
**H3K27Ac** Abcam (ab4729)	Dako pH6 20'@100°C	Sniper + BSA 10mins	1:250 DVG 45 min	Mach2 Rabbit HRP 15 mins	Opal 570 10mins
**H3K4me2** Abcam (ab32356)	Dako pH6 20'@100°C	Sniper + BSA 10mins	1:100 DVG 60 min	Goat on Rodent probe and polymer 10+10 min	Opal 620 10mins
**KRT5** Assay Matrix (100312-T38)	Dako pH9.0 20'@100°C	Sniper/BSA 10 min	1:1000 30 min	MACH2 Rabbit HRP 15 min	Opal 690 10 min
**Muc5A** Assay Matrix (102400-T08)	Dako pH9.0 5'@125°C	Sniper + BSA 10 min	1:1000 30 min	MACH 1 Mouse Probe 15' Poly 15'	Opal 570 10 min
**PanCK** Dako (M3515)	Dako pH 9.0 20'@100°C	Sniper/BSA 10 min	1:5000 VGY 30 min	PE Mouse HRP 15 min	Opal 570 10 min
**Perforin** Santa Cruz (sc136994)	Dako pH 9.0 15'@105°C	Sniper + BSA 10 min	1:100 DVG 30 min	PE Mouse HRP 15 min	Opal 520 10 min
**SARS-CoV-2** Sino Biological (40150-R007)	Dako pH9.0 20'@100°C	Sniper + BSA 10 min	1:1000 DVG 30 min	MACH2 Rabbit HRP 15 min	Opal 520 10 min

Supplementary Table 3. PBMC staining conditions.

Antibody	Antigen Ret.	Blocking	Primary	Secondary	TSA
**ACE2** [me] Rao custom antibody	Permeabilize with 0.1% Triton X-100 20 min	Sniper/BSA 10 min	1:500 DVG 60 min	MACH2 Rabbit HRP 20 min	Opal 650 15 min
**ACE-2** R&D Systems (AF-933)	Permeabilize with 0.1% Triton X-100 20 min or biocare denaturing min no AR	Sniper + BSA 10 min	1:500 DVG 60 min	MACH2 Rabbit HRP 20 min	Opal 650 15 min
**CD14** Abcam (ab133335)	Biocare Medical Denaturing Solution 5 min No AR	Sniper/BSA 10 min	1:2000 DVG 30 min	PE Rabbit HRP 15 min	Opal 520 10 min
**IL-6 (10C12)** Novocastra (NCL-L-IL6)	Biocare Medical Denaturing Solution 5 min No AR	Sniper/BSA 10 min	1:500 VGY 30 min	PE Mouse HRP 15 min	Opal 570 10 min
**H3K27Ac** Abcam (ab4729)	Dako pH6 20'@100°C	Sniper + BSA 10 min	1:250 DVG 45 min	Mach2 Rabbit HRP 15 min	Opal 570 10 min
**H3K4me2** Abcam (ab32356)	Dako pH6 20'@100°C	Sniper + BSA 10 min	1:100 DVG 60 min	BC Goat on Rodent Probe and Polymer 10+10 min	Opal 620 10 min

Supplementary Table 4. Antibodies used in cell line studies.

Antibody	Antigen Ret.	Blocking	Primary	TSA
**ACE2**<br>Abcam (ab15348)	Permeabilize with 0.1% Triton X-100 20 min	1% BSA<br>30 min	1:100<br>120 min	Duolink or CoIP
**IMPα**<br>Santa Cruz (sc101292)	Permeabilize with 0.1% Triton X-100 20 min	1% BSA<br>30 min	1:100<br>120 min	Duolink or WB
**ACE2^{me}**<br>Rao custom antibody	Permeabilize with 0.1% Triton X-100 20 min	1% BSA<br>30 min	1:100<br>120 min	WB
**c-Rel** (B-6)<br>Santa Cruz (sc-6955)	Permeabilize with 0.5% Triton X-100, 15 min	1% BSA<br>30 min	1:100<br>60 min	Opal 568<br>10 min
**PKC zeta** (phospho T560)<br>Abcam (ab62372)	Permeabilize with 0.5% Triton X-100, 15 min	1% BSA<br>30 min	1:100<br>60 min	Opal 488<br>10 min
**HDAC1**<br>prosci-inc (42-976)	Permeabilize with 0.5% Triton X-100, 15 min	1% BSA<br>30 min	1:100<br>60 min	Opal 647<br>10 min
**NFκB p50** (C-19)<br>Santa Cruz (sc-1190)	Permeabilize with 0.5% Triton X-100, 15 min	1% BSA<br>30 min	1:100<br>60 min	Opal 647<br>10 min
**NFATc1** (7A6)<br>Santa Cruz (sc-7294)	Permeabilize with 0.5% Triton X-100, 15 min	1% BSA<br>30 min	1:100<br>60 min	Opal 568<br>10 min
**Nurr1/Nurr77**<br>US biological life sciences (N6889-95A2B)	Permeabilize with 0.5% Triton X-100, 15 min	1% BSA<br>30 min	1:100<br>60 min	Opal 488<br>10 min
**ACE2** R&D Systems (AF-933)	Permeabilize with 0.5% Triton X-100, 15 min	1% BSA<br>30 min	1:100<br>60 min	Opal 650<br>15 min

Supplementary Table 5. Antibodies used in flow cytometry.

Antibody	Catalogue No.	Company	Dilution
**ACE2** (AF594)	sc390851	Santa Cruz	1:100
**ACE2me** (Rao custom)		Mimotopes	1:100
**CD45** (PerCP/Cy5.5)	304027	BioLegend	1:200
**CD3** (BV421)	562426	BD Biosciences	1:200
**CD14** (AF700)	561029	BD Biosciences	1:200
Rabbit IgG isotype control	31235	Thermo Fisher	1:100
Secondary donkey anti-rabbit (AF647)	A31573	Thermo Fisher	1:500
LIVE/DEAD™ Fixable Near IR (780) Viability kit	L34992	Thermo Fisher	1:1000

Uncropped NanoString and IF pictures

Figure 4c

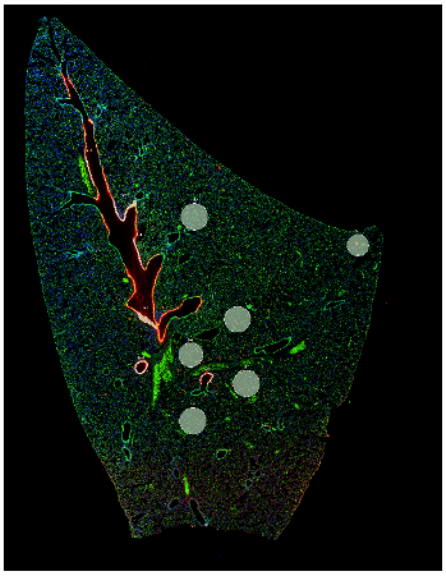

Nanostring image

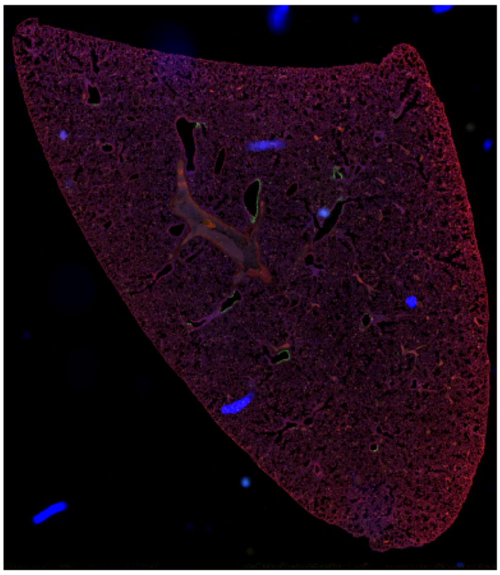

IF image

Uncropped western blots

Supplementary Figure 1a

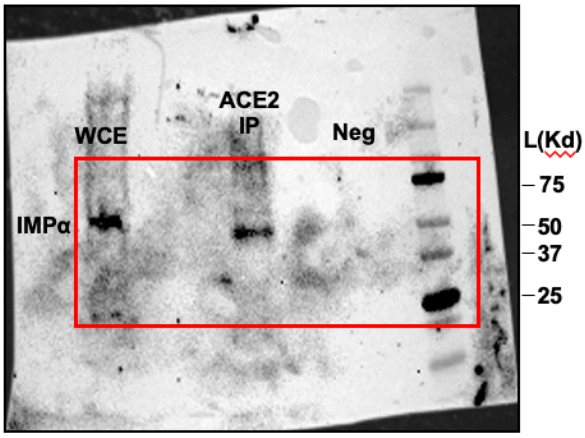

Antibodies used:
Rabbit anti-ACE2
Ab15348, Abcam

Mouse anti-IMPα
sc-101292, Santa Cruz Biotechnology

Supplementary Figure 4b

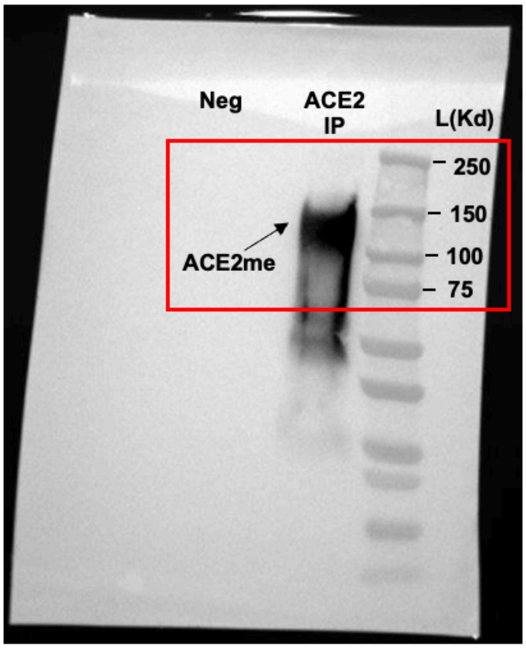

Antibodies used:
Rabbit anti-ACE2
Ab15348, Abcam

Rabbit anti-ACE2^{me}
Rao custom antibody
