# Supplementary material for: In vivo inhibition of nuclear ACE2 translocation protects against SARS-CoV-2 replication and lung damage through epigenetic imprinting
Source: Nat Commun. 2023 Jun 27;14:3680. doi: 10.1038/s41467-023-39341-4 (PMC10300102; doi:10.1038/s41467-023-39341-4)
Supplement: Supplementary file 2 — Reporting Summary [file 41467_2023_39341_MOESM2_ESM.pdf]

# nature portfolio

Corresponding author(s): Sudha Rao

Last updated by author(s): May 18, 2023



# Reporting Summary

Nature Portfolio wishes to improve the reproducibility of the work that we publish. This form provides structure for consistency and transparency in reporting. For further information on Nature Portfolio policies, see our Editorial Policies and the Editorial Policy Checklist.

## Statistics

For all statistical analyses, confirm that the following items are present in the figure legend, table legend, main text, or Methods section.

| n/a | Confirmed | |
|---|---|---|
| ☐ | ☒ | The exact sample size ($n$) for each experimental group/condition, given as a discrete number and unit of measurement |
| ☐ | ☒ | A statement on whether measurements were taken from distinct samples or whether the same sample was measured repeatedly |
| ☐ | ☒ | The statistical test(s) used AND whether they are one- or two-sided<br>*Only common tests should be described solely by name; describe more complex techniques in the Methods section.* |
| ☒ | ☐ | A description of all covariates tested |
| ☐ | ☒ | A description of any assumptions or corrections, such as tests of normality and adjustment for multiple comparisons |
| ☐ | ☒ | A full description of the statistical parameters including central tendency (e.g. means) or other basic estimates (e.g. regression coefficient) AND variation (e.g. standard deviation) or associated estimates of uncertainty (e.g. confidence intervals) |
| ☐ | ☒ | For null hypothesis testing, the test statistic (e.g. $F$, $t$, $r$) with confidence intervals, effect sizes, degrees of freedom and $P$ value noted<br>*Give P values as exact values whenever suitable.* |
| ☒ | ☐ | For Bayesian analysis, information on the choice of priors and Markov chain Monte Carlo settings |
| ☒ | ☐ | For hierarchical and complex designs, identification of the appropriate level for tests and full reporting of outcomes |
| ☐ | ☒ | Estimates of effect sizes (e.g. Cohen's $d$, Pearson's $r$), indicating how they were calculated |

*Our web collection on statistics for biologists contains articles on many of the points above.*

## Software and code

Policy information about availability of computer code

| | |
|---|---|
| Data collection | All software used to perform data collection are described in the Methods or Supplementary Information. Flow cytometry data were collected using BD FACSDiva (version 8.01). |
| Data analysis | All sources of code used to perform DSP and integrative bioinformatics analyses are described in the Methods. Flow cytometry data were analyzed using FlowJo (BD, version 10.7.1). Statistical analyses were performed in Prism (GraphPad, version 9.3.1). NovaSeq 6000 FASTQ files were processed using the NanoString DND pipeline (Nanostring, v2.4.0.146) and NLStradamus prediction Revision r.9 (http://www.moseslab.csb.utoronto.ca/NLStradamus). R packages: Rtsne, lme4, fgsea, ReactomePA, SpatialDecon were used for differential gene expression, enrichment, and cell type analysis. The raw counts from the QC-qualified ROIs were normalized using third quartile (Q3) normalization factors. For each sample, the LoQ (limit of quantitation) was calculated using the geometric mean of the negative probes multiplied by the geometric standard deviation of negative probes raised to a power of 2. We removed genes below LoQ in all ROIs. QuPath (v0.4.3) was used for cell segmentation from OME-tiff imaging data and the Python package SimpleITK was used for image registration. Differential gene expression, enrichment, and cell type composition differences were tested. |

For manuscripts utilizing custom algorithms or software that are central to the research but not yet described in published literature, software must be made available to editors and reviewers. We strongly encourage code deposition in a community repository (e.g. GitHub). See the Nature Portfolio guidelines for submitting code & software for further information.

## Data

Policy information about availability of data

All manuscripts must include a data availability statement. This statement should provide the following information, where applicable:

- Accession codes, unique identifiers, or web links for publicly available datasets
- A description of any restrictions on data availability
- For clinical datasets or third party data, please ensure that the statement adheres to our policy

The data supporting the findings of this work are available within the paper and in the Supplementary Information file. Source data are provided as a source data file. Source data are provided with this paper.

## Human research participants

Policy information about studies involving human research participants and Sex and Gender in Research.

| | |
|---|---|
| Reporting on sex and gender | No subgroup analyses on sex and gender due to the small sample size and exploratory design |
| Population characteristics | COVID-19 patients:<br>Whole blood samples were collected from COVID-19 patients (total n=24, mild n=11, moderate n=7, or severe n=6 according to the WHO seven-point ordinal scale) admitted to the Royal Brisbane and Women's Hospital (RBWH) processed within 4 h of collection. Patients were aged from 29 to 68 years of age, with 7 males and 17 females.<br>Infected (convalesced) patients:<br>The inclusion criteria for convalescent participants was that they exceed 18 years of age, had been clinically diagnosed by PCR with SARS-CoV-2 infection, and had subsequently resolved symptomatic infection and been released from isolation under ethical approval P3618 (QIMR Berghofer Medical Research Institute Human Research Ethics Committee). PBMC were isolated from convalescent participants at a median of 58 days post infection. Patients were aged between 20 and 65 years of age.<br>Vaccinated patients:<br>PBMC samples were taken from: 1) n=14 participants prior to vaccination (PRE) and four weeks after a second vaccination (POST, Pfizer or Astra-Zeneca), who were further grouped into individuals with (n=7) and without (n=7) subsequent infection; 2) n=5 participants at four weeks after 3rd COVID-19 vaccine (PB, Pfizer or Astra-Zeneca) and four weeks post-infection (PI), following recovery from a positive COVID-19 rapid antigen test. Donors were aged between 34 and 63 years of age. 15% of donors had received the Pfizer vaccination and 85% the AstraZeneca vaccination. |
| Recruitment | The QIMR Berghofer external relations team issued a public recruitment call for relevant participants. Donor SARS-CoV-2 status was confirmed by PCR and all donors were in the convalescent stage. Whole blood samples were collected from all participants after obtaining written informed consent.<br><br>The recruitment of these patients did not introduce any bias in this study. |
| Ethics oversight | COVID-19 patients:<br>This study was performed in accordance with the NHMRC National Statement on Ethical Conduct in Human Research 2007 (updated 2018). Ethical approval was granted by the Royal Brisbane and Women's Hospital Ethics Committee (HREC/2020/QRBW/63138) and accepted by the QIMR Berghofer (P3562).<br><br>Infected (convalesced) patients and vaccinated patients:<br>Patients had been released from isolation under ethical approval P3618 (QIMR Berghofer Medical Research Institute Human Research Ethics Committee). |

Note that full information on the approval of the study protocol must also be provided in the manuscript.

# Field-specific reporting

Please select the one below that is the best fit for your research. If you are not sure, read the appropriate sections before making your selection.

- [x] Life sciences
- [ ] Behavioural & social sciences
- [ ] Ecological, evolutionary & environmental sciences

For a reference copy of the document with all sections, see nature.com/documents/nr-reporting-summary-flat.pdf

# Life sciences study design

All studies must disclose on these points even when the disclosure is negative.

| | |
|---|---|
| Sample size | Animal<br>For animal studies, n=3/group was chosen for the tolerance study due to animal availability, and n=8/group was chosen for efficacy studies. No sample size calculations were used as sample size for animal studies were determined based upon previous experience with similar studies (PMID: 31249575) where up to 5-8 animals per group represented a sufficient sample size to detect statistical differences between experimental groups.<br><br>Human<br>The human study was exploratory and correlative with the basic science studies, and the sample size was selected based on sample availability. |
| Data exclusions | No data excluded. |
| Replication | All experimental data were reliably generated at least with n=3 animals/group or n=2 human participants. Details of independent biological and technical replicates are described in the figure legends for each experimental assay. |
| Randomization | Animals were randomly assigned to each experimental groups. The patients were allocated based on the criteria of the cohort recruitment. |
| Blinding | Investigators were blinded to group allocation during data collection and and analysis. Additionally, all pathology analysis carried out on the hamster model was blinded. |

# Reporting for specific materials, systems and methods

We require information from authors about some types of materials, experimental systems and methods used in many studies. Here, indicate whether each material, system or method listed is relevant to your study. If you are not sure if a list item applies to your research, read the appropriate section before selecting a response.

## Materials & experimental systems

| n/a | Involved in the study | |
|---|---|---|
| ☐ | ☒ | Antibodies |
| ☐ | ☒ | Eukaryotic cell lines |
| ☒ | ☐ | Palaeontology and archaeology |
| ☐ | ☒ | Animals and other organisms |
| ☒ | ☐ | Clinical data |
| ☒ | ☐ | Dual use research of concern |

## Methods

| n/a | Involved in the study | |
|---|---|---|
| ☒ | ☐ | ChIP-seq |
| ☐ | ☒ | Flow cytometry |
| ☒ | ☐ | MRI-based neuroimaging |

## Antibodies

| | |
|---|---|
| Antibodies used | Rabbit anti-SARS-COV-2 Spike (1:1000, Sino Biological, 40150-R007), Rabbit anti-ACE2 (1:500, R&D systems, AF-933), Rabbit anti-ACE2 (1:1000, Abcam, ab15348), mouse anti-IMPα (1:100 Santa Cruz, sc-101292), Mouse anti-PanCK-488 at (1:5000, Dako M3515), mouse Perforin 1 (F-1) 1:100, Santa Cruz Sc-136994), rabbit anti CD3 (1:3000, Dako A0452), rat anti F4/80 (CI:A3-1) 1:4000 abcam ab6640 (4.21), mouse anti CD14 1:200 abcam ab181470, mouse anti IL-6 (10C12) 1:500 Novocastra NCL-L-IL6, rabbit anti-KRT5 (1:1000, Assay Matrix, 100312-T38), mouse anti-Muc5A (1:1000, Assay Matrix, 102400-T08), rabbit anti-Histone H3 (acetyl K27) antibody - ChIP Grade 1:250 abcam ab4729, rabbit anti-Histone H3 (di methyl K4) antibody [Y47] - ChIP Grade 1:100 abcam ab32356, rabbit anti-CD14 antibody [EPR3653] 1:2000 abcam ab133335, mouse anti Anti-c-Rel Antibody (B-6): 1:100 Santa Cruz, sc-6955, rabbit Anti-PKC zeta (phospho T560) antibody [EP2037AY] 1:100 abcam ab62372, goat anti HDAC1 Antibody, 1:100 prosci-inc 42-976, goat anti-NFκB p50 Antibody (C-19): 1:100 Santa Cruz sc-1190, mouse Anti-NFATc1 Antibody (7A6): 1:100 Santa Cruz sc-7294, rabbit Anti-Nuclear Receptor Subfamily 4, Group A, Member 2 Antibody (Nurr1/Nurr77) 1:100 US biological life sciences N6889-95A2B, Donkey anti-Goat IgG (H+L) Cross-Adsorbed Secondary Antibody, Alexa Fluor 647, 1/500, Thermofisher Scientific A21447, Donkey anti-Mouse IgG (H+L) Highly Cross-Adsorbed Secondary Antibody Alexa Fluor 568, 1/500, Thermofisher Scientific A10037, Donkey anti-Rabbit IgG (H+L) Highly Cross-Adsorbed Secondary Antibody, Alexa Fluor 488 1/500, Thermofisher Scientific A21206, Donkey anti-rabbit AF568 (A10042, Thermo Fisher Scientific). Opal Tyramide (TSA) 6-Plex Manual Detection Kit Akoya Biosciences NEL821001KT, Custom antibodies targeting ACE2me and ACE2unmod (Mimotopes). FACS antibodies: Primary antibodies targeting ACE2me (custom), secondary donkey anti-rabbit AF647 (A31573, Thermo Fisher Scientific), ACE2 (sc390851, Santa Cruz Biotechnology, Dallas, TX), CD45 (304027, BioLegend, San Diego, CA), CD3 (562426, BD Biosciences, San Jose, CA), and CD14 (561029, BD Biosciences). LIVE/DEAD Fixable Near IR (780) viability kit (L34992, Thermo Fisher Scientific). |
| Validation | All commercial primary antibodies were validated by the manufacturer. Species and application validations for primary antibodies can be found in the antibody datasheets available at the following locations:<br>Rabbit anti-SARS-COV-2 Spike (1:1000, Sino Biological, 40150-R007): https://www.sinobiological.com/antibodies/cov-spike-40150- |

r007
Rabbit  anti-ACE2 (1:500, R&D systems, AF-933): https://www.rndsystems.com/products/human-mouse-rat-hamster-ace-2-antibody_af933
Rabbit anti-ACE2 (1:1000, Abcam, ab15348): https://www.abcam.com/products/primary-antibodies/ace2-antibody-ab15348.html
mouse anti-IMPα (1:100 Santa Cruz, sc-101292)
https://www.scbt.com/p/karyopherin-alpha1-antibody-187-1
Mouse anti-PanCK-488 at (1:5000, Dako M3515): https://www.agilent.com/en/product/immunohistochemistry/antibodies-controls/primary-antibodies/cytokeratin-(concentrate)-76562
Mouse Perforin 1 (F-1) 1:100, Santa Cruz Sc-136994): https://www.scbt.com/p/perforin-1-antibody-f-1
rabbit anti CD3 (1:4000, Dako A0452)
https://www.agilent.com/en/product/immunohistochemistry/antibodies-controls/primary-antibodies/cd3-(concentrate)-76133
F4/80 (CI:A3-1) 1/4000 abcam ab6640 (4.21):
https://www.abcam.com/f480-antibody-cia3-1-macrophage-marker-ab6640.html
mouse anti CD14 1/200 abcam ab181470
https://www.abcam.com/cd14-antibody-1h5d8-ab181470.html
mouse anti IL-6 (10C12) 1:200 Novocastra NCL-L-IL6
https://shop.leicabiosystems.com/en-au/ihc-ish/ihc-primary-antibodies/pid-interleukin-6
rabbit anti-KRT5 (1:1000, Assay Matrix, 100312-T38)
https://www.sinobiological.com/antibodies/human-cytokeratin-5-krt5-100312-t38
mouse anti-Muc5A (1:1000, Assay Matrix, 102400-T08)
https://www.sinobiological.com/antibodies/human-muc5a-102400-t08
Rabbit anti-Histone H3 (acetyl K27) antibody - ChIP Grade 1/200 abcam ab4729
https://www.abcam.com/histone-h3-acetyl-k27-antibody-chip-grade-ab4729.html
Rabbit Anti-Histone H3 (di methyl K4) antibody [Y47] - ChIP Grade 1/200 abcam ab32356
https://www.abcam.com/histone-h3-di-methyl-k4-antibody-y47-chip-grade-ab32356.html
rabbit anti-CD14 antibody [EPR3653] 1/2000 abcam ab133335
https://www.abcam.com/cd14-antibody-epr3653-ab133335.html
mouse anti Anti-c-Rel Antibody (B-6): 1/200 Santa Cruz, sc-6955
https://www.scbt.com/p/c-rel-antibody-b-6
rabbit Anti-PKC zeta (phospho T560) antibody [EP2037AY] 1:200 abcam ab62372
https://www.abcam.com/pkc-zeta-phospho-t560-antibody-ep2037ay-ab62372.html
goat anti HDAC1 Antibody, 1:200 prosci-inc 42-976.
https://www.prosci-inc.com/product/hdac1-antibody-42-976/
goat anti-NFκB p50 Antibody (C-19): 1:200 Santa Cruz sc-1190
https://www.scbt.com/p/nfkappab-p50-antibody-c-19
mouse Anti-NFATc1 Antibody (7A6): 1:100 Santa Cruz sc-7294
https://www.scbt.com/p/nfatc1-antibody-7a6
rabbit Anti-Nuclear Receptor Subfamily 4, Group A, Member 2 Antibody (Nurr1/Nurr77) 1:100 US biological life sciences N6889-95A2B,
https://www.usbio.net/antibodies/N6889-95A2B/Nuclear-Receptor-Subfamily-4-Group-A-Member-2-NR4A2-HZF3-Immediateearly-Response-Protein-NOT-Intermediate-Early-Receptor-Protein-NGFIBnur77-beta-Type-Transcription-Factor-Homolog-NOT-Nur-Related-Protein-1-Homolog-Nurr1-Orphan-Nuclear-Receptor-NR4A2-Orphan-Nuclear-Receptor-NURR1-RNR1-T-cell-Nuclear-Receptor-NOT-Transcriptionally-Inducible-Nuclear-Receptor-TINUR-Transcriptionally-Inducible-Nuclear-Receptor-Related
Donkey anti-Goat IgG (H+L) Cross-Adsorbed Secondary Antibody, Alexa Fluor 647, 1/500, Thermofisher Scientific A21447,
https://www.thermofisher.com/antibody/product/A-21447
Donkey anti-rabbit AF568 (A10042, Thermo Fisher Scientific). https://www.thermofisher.com/antibody/product/A10042,
Donkey anti-Mouse IgG (H+L) Highly Cross-Adsorbed Secondary Antibody Alexa Fluor 568, 1/500, Thermofisher Scientific A10037,
https://www.thermofisher.com/antibody/product/A10037
Donkey anti-Rabbit IgG (H+L) Highly Cross-Adsorbed Secondary Antibody, Alexa Fluor 488 1/500, Thermofisher Scientific A21206,
https://www.thermofisher.com/antibody/product/A21206
Opal Tyramide (TSA) 6-Plex Manual Detection Kit Akoya Biosciences NEL821001KT
https://www.akoyabio.com/phenoimager/assays/opal-7-color-ihc-kit/
ACE2 (sc390851, Santa Cruz Biotechnology, Dallas, TX),
https://www.scbt.com/p/ace2-antibody-e-11
CD45 (304027, BioLegend, San Diego, CA),
https://www.biolegend.com/en-us/products/percp-cyanine5-5-anti-human-cd45-antibody-4240?GroupID=BLG5926
CD3 (562426, BD Biosciences, San Jose, CA),
https://www.bdbiosciences.com/en-us/products/reagents/flow-cytometry-reagents/research-reagents/single-color-antibodies-ruo/bv421-mouse-anti-human-cd3.562426
CD14 (561029, BD Biosciences, San Jose, CA),
https://www.bdbiosciences.com/en-ca/products/reagents/flow-cytometry-reagents/research-reagents/single-color-antibodies-ruo/alexa-fluor-700-mouse-anti-human-cd14.561029
LIVE/DEAD™ Fixable Near IR (780) Viability Kit
https://www.thermofisher.com/order/catalog/product/L34992

For custom antibodies:
Custom polyclonal rabbit antibodies targeting ACE2 sequence QAKTFLD(Kme)FNHEAED were generated by Mimotopes (Mulgrave, Victoria, Australia). Briefly, for antibody generation, a cysteine was incorporated at the C-terminus of the peptide (peptide sequence CQAKTFLD(Kme)FNHEAED) and reacted to conjugate the peptide to an immunogenic carrier protein keyhole limpet hemocyanin

(KLH). No special immunization protocols were required to generate anti-methylated peptide antibodies. Rabbits were immunized several weeks apart. The first immunization was with an emulsion of the peptide conjugate with complete Freund's adjuvant and the second using incomplete Freund's adjuvant. Potent anti-peptide sera were obtained after several weeks. Methylated/unmodified-peptide antisera were conveniently tested by ELISA, where the sera were titrated on microtiter plates coated with non-methylated peptide and methylated peptide. For methylated peptide antibody enhancement, the non-methylated analog of the peptide used for the immunization was coupled to a gel Sulfo Link (20401:05273 Thermo Fisher Scientific, Waltham, MA) using the available cysteine residue following the manufacturer's instructions. The resultant gel was incubated with aliquots of the antisera to absorb antibodies specific to the non-methylated peptide. The resultant antiserum had enhanced specificity for the methylated peptide sequence. To produce affinity-purified antibodies specific to the methylated peptide only, it was necessary to first perform enhancement to remove antibodies from the serum targeting the non-methylated peptide. Specificity of the affinity-purified antibodies was tested by ELISA using both the non-methylated and the methylated peptides coated onto the plate. ELISA results are depicted in Supplementary Fig. 3a,b and show antibody specificity for their targets with appropriate blank/negative controls demonstrating no signals. The ELISA also shows that the antibody specific for the methylated target does not recognize the unmodified peptide and vice versa and is therefore specific for the methylated target. This approach used blanks and negative controls in the ELISA to show that the antibody signals were not background and specific for the target peptide. To further confirm antibody specificity the ACE2me and CD14 antibody mix was pre incubated with or without blocking peptides specific for custom antibody target lysine 31 sequence. As expected, blocking peptides only blocked signal for the custom ACE2me antibody and did not affect the CD14 staining as depicted in Supplementary Fig. 3b.

## Eukaryotic cell lines

Policy information about cell lines and Sex and Gender in Research

| | |
|---|---|
| Cell line source(s) | Caco-2 (human epithelial colorectal adenocarcinoma) cells (ATCC Cat # HTB-37™), MRC-5 (human fetal lung fibroblast) cells (ATCC Cat#CCL-171™), NCI-H1299 (ATCC Cat#CRL-5803™), VERO C1008 (ATCC Cat#CRL-1586). |
| Authentication | ATCC cell lines were authenticated by ATCC. |
| Mycoplasma contamination | Cell line tested negative for Mycoplasma contamination |
| Commonly misidentified lines (See ICLAC register) | No commonly misidentified cell lines used in the study |

## Animals and other research organisms

Policy information about studies involving animals; ARRIVE guidelines recommended for reporting animal research, and Sex and Gender in Research

| | |
|---|---|
| Laboratory animals | Female Golden Syrian hamsters (6-8 weeks obtained from Janiver Labs, France) and Female/Male C57BL/6J mice (6-8 weeks obtained from the Animal Resources Centre, Australia). Hamsters were maintained in housing rooms under controlled environmental conditions: temperature: 22 ± 2°C, humidity 55 ± 10%, photoperiod (12h light/12h dark), F9 filtered air (CFH), minimum of 12 air exchanges per hour with no recirculation. Each cage is labeled with a specific code. Animal enclosures provide sterile and adequate space with bedding material, food and water, environmental and social enrichment (group housing). Mice were maintained in housing rooms under controlled environmental conditions: individually ventilated cages (IVCs) with a 12/12 light/dark cycle. The ambient temperature is set to 20-25°C and relative humidity at 30-70% rH. |
| Wild animals | No wild animals were involved in the study |
| Reporting on sex | Sex-based analyses is not relevant to the study. |
| Field-collected samples | No field-collected samples were involved in the study |
| Ethics oversight | The hamster study was conducted according to the French and European Regulations and the National Research Council Guide for the Care and Use of Laboratory Animals. All animal procedures used were submitted and approved by the Institutional Animal Care and Use Committee of Oncodesign® Biotechnology approved by French authorities. Project approval reference D9203202.<br>The mice study was approved by the University of Queensland Animal Ethics Committee. Project code 2021/AE000659. |

Note that full information on the approval of the study protocol must also be provided in the manuscript.

## Flow Cytometry

### Plots

Confirm that:

- [x] The axis labels state the marker and fluorochrome used (e.g. CD4-FITC).
- [x] The axis scales are clearly visible. Include numbers along axes only for bottom left plot of group (a 'group' is an analysis of identical markers).
- [x] All plots are contour plots with outliers or pseudocolor plots.
- [x] A numerical value for number of cells or percentage (with statistics) is provided.

## Methodology

| | |
|---|---|
| Sample preparation | Blood samples were processed for PBMC isolation within 4 hours of collection and stored at -80° until use. Isolated PBMCs were thawed and washed with D-PBS and resuspended in PBS containing 1% BSA prior to antibody staining against custom primary polyclonal rabbit antibody ACE2me (Mimotopes, Mulgrave, Victoria, Australia), secondary donkey anti-rabbit AF647 (A31573, Thermo Fisher Scientific), ACE2 (sc390851, Santa Cruz Biotechnology, Dallas, TX), CD45 (304027, BioLegend, San Diego, CA), CD3 (562426, BD Biosciences, San Jose, CA), and CD14 (561029, BD Biosciences). LIVE/DEAD Fixable Near IR (780) viability kit (L34992, Thermo Fisher Scientific). |
| Instrument | BD LSRFortessa Flow Cytometer (BD Biosciences, Franklin Lakes, NJ) |
| Software | BD FACSDiva (version 8.01) and FlowJo (version 10.7.1) |
| Cell population abundance | N/A, no cell sorting was required in the study |
| Gating strategy | CD14+ACE2me+: FSC-A/SSC-A > single cell FSC-A/FSC-H > viable cell FSC-A/LIVE_DEAD Fixable > CD45pos > CD3neg/CD14pos > ACE2me pos<br><br>CD14+ACE2+: FSC-A/SSC-A > single cell FSC-A/FSC-H > viable cell FSC-A/LIVE_DEAD Fixable > CD45pos > CD3neg/CD14pos > ACE2 pos<br><br>CD3+ACE2me+: FSC-A/SSC-A > single cell FSC-A/FSC-H > viable cell FSC-A/LIVE_DEAD Fixable > CD45pos > CD3pos/CD14neg > ACE2me pos<br><br>CD3+ACE2+: FSC-A/SSC-A > single cell FSC-A/FSC-H > viable cell FSC-A/LIVE_DEAD Fixable > CD45pos > CD3pos/CD14neg > ACE2 pos |

☒ Tick this box to confirm that a figure exemplifying the gating strategy is provided in the Supplementary Information.
